# Effectiveness of non-pharmaceutical interventions as implemented in the UK during the COVID-19 pandemic: a rapid review

**DOI:** 10.1093/pubmed/fdaf017

**Published:** 2025-03-04

**Authors:** T Ashcroft, E McSwiggan, E Agyei-Manu, M Nundy, N Atkins, J R Kirkwood, M Ben Salem Machiri, V Vardhan, B Lee, E Kubat, S Ravishankar, P Krishan, U De Silva, E O Iyahen, J Rostron, A Zawiejska, K Ogarrio, M Harikar, S Chishty, D Mureyi, B Evans, D Duval, S Carville, S Brini, J Hill, M Qureshi, Z Simmons, I Lyell, T Kavoi, M Dozier, G Curry, J M Ordóñez-Mena, S de Lusignan, A Sheikh, E Theodoratou, R McQuillan

**Affiliations:** Usher Institute, Centre for Global Health, University of Edinburgh, Edinburgh EH16 4UX, UK; Usher Institute, Centre for Population Health Sciences, University of Edinburgh, Edinburgh EH16 4UX, UK; Usher Institute, Centre for Global Health, University of Edinburgh, Edinburgh EH16 4UX, UK; Usher Institute, Centre for Global Health, University of Edinburgh, Edinburgh EH16 4UX, UK; Usher Institute, Centre for Global Health, University of Edinburgh, Edinburgh EH16 4UX, UK; Usher Institute, Centre for Global Health, University of Edinburgh, Edinburgh EH16 4UX, UK; Usher Institute, Centre for Medical Informatics, University of Edinburgh, Edinburgh EH16 4UX, UK; Usher Institute, Centre for Global Health, University of Edinburgh, Edinburgh EH16 4UX, UK; Usher Institute, Centre for Global Health, University of Edinburgh, Edinburgh EH16 4UX, UK; Usher Institute, Centre for Global Health, University of Edinburgh, Edinburgh EH16 4UX, UK; Usher Institute, Centre for Global Health, University of Edinburgh, Edinburgh EH16 4UX, UK; Usher Institute, Centre for Global Health, University of Edinburgh, Edinburgh EH16 4UX, UK; Usher Institute, Centre for Global Health, University of Edinburgh, Edinburgh EH16 4UX, UK; Usher Institute, Centre for Global Health, University of Edinburgh, Edinburgh EH16 4UX, UK; Usher Institute, Centre for Global Health, University of Edinburgh, Edinburgh EH16 4UX, UK; Usher Institute, Centre for Global Health, University of Edinburgh, Edinburgh EH16 4UX, UK; Usher Institute, Centre for Global Health, University of Edinburgh, Edinburgh EH16 4UX, UK; Usher Institute, Centre for Global Health, University of Edinburgh, Edinburgh EH16 4UX, UK; School of Public Health and Tropical Medicine—Department of Social, Behavioral, and Population Sciences, Tulane University, New Orleans, LA 70112, USA; Usher Institute, Centre for Global Health, University of Edinburgh, Edinburgh EH16 4UX, UK; Usher Institute, Centre for Global Health, University of Edinburgh, Edinburgh EH16 4UX, UK; Usher Institute, Centre for Global Health, University of Edinburgh, Edinburgh EH16 4UX, UK; Science Evidence Review Team, Research, Evidence and Knowledge Division, UKHSA, London E14 4PU, UK; Science Evidence Review Team, Research, Evidence and Knowledge Division, UKHSA, London E14 4PU, UK; Clinical and Public Health Response Evidence Review Team, Clinical and Public Health, UKHSA, London E14 4PU, UK; Clinical and Public Health Response Evidence Review Team, Clinical and Public Health, UKHSA, London E14 4PU, UK; Clinical and Public Health Response Evidence Review Team, Clinical and Public Health, UKHSA, London E14 4PU, UK; Clinical and Public Health Response Evidence Review Team, Clinical and Public Health, UKHSA, London E14 4PU, UK; Science Evidence Review Team, Research, Evidence and Knowledge Division, UKHSA, London E14 4PU, UK; Health Protection Operation, UKHSA, London E14 4PU, UK; Clinical and Public Health Response Evidence Review Team, Clinical and Public Health, UKHSA, London E14 4PU, UK; Information Services, University of Edinburgh, Edinburgh EH3 9DR, UK; Usher Institute, Centre for Population Health Sciences, University of Edinburgh, Edinburgh EH16 4UX, UK; Nuffield Department of Primary Care Health Sciences, University of Oxford, Oxford OX2 6GG, UK; Nuffield Department of Primary Care Health Sciences, University of Oxford, Oxford OX2 6GG, UK; Royal College of General Practitioners (RCGP), Research and Surveillance Centre, London NW1 2FB, UK; Usher Institute, Centre for Medical Informatics, University of Edinburgh, Edinburgh EH16 4UX, UK; Nuffield Department of Primary Care Health Sciences, University of Oxford, Oxford OX2 6GG, UK; Usher Institute, Centre for Global Health, University of Edinburgh, Edinburgh EH16 4UX, UK; Usher Institute, Centre for Global Health, University of Edinburgh, Edinburgh EH16 4UX, UK

**Keywords:** COVID-19, non-pharmaceutical interventions

## Abstract

**Background:**

Although non-pharmaceutical inventions (NPIs) were used globally to control the spread of COVID-19, their effectiveness remains uncertain. We aimed to assess the evidence on NPIs as implemented in the UK, to allow public health bodies to prepare for future pandemics.

**Methods:**

We used rapid systematic methods (search date: January 2024) to identify, critically appraise and **synthesize** interventional, observational and modelling studies reporting on NPI effectiveness in the UK.

**Results:**

Eighty-five modelling, nine observational and three interventional studies were included. Modelling studies had multiple quality issues; six of the 12 non-modelling studies were high quality. The best available evidence was for test and release strategies for case contacts (moderate certainty), which was suggestive of a protective effect. Although evidence for school-related NPIs and universal lockdown was also suggestive of a protective effect, this evidence was considered low certainty. Evidence certainty for the remaining NPIs was very low or inconclusive.

**Conclusion:**

The validity and reliability of evidence on the effectiveness of NPIs as implemented in the UK during the COVID-19 pandemic is weak. To improve evidence generation and support decision-making during future pandemics or other public health emergencies, it is essential to build evaluation into the design of public health interventions.

## Introduction

Non-pharmaceutical interventions (NPIs) are measures not dependent on medication that employ mitigation and suppression strategies to reduce case numbers to low levels.^1^^,^^2^ During the first year of the COVID-19 pandemic, NPIs were the only preventive methods available to governments, health systems and populations.^3^ In the UK, as in other jurisdictions, NPIs were mainly implemented as a combination of measures. Reviews from around the world suggest that this approach is likely to have been effective,^4–7^ with observational data from Hong Kong, New Zealand and South Korea, suggesting sustained suppression of the virus.^8^ However, there was considerable variation across the world in how, when and in what combination NPIs were implemented, making it challenging to assess the effectiveness of individual measures or generalize this evidence to specific contexts.^2^^,^^9^^,^^10^ Therefore, there is a need to assess the effectiveness of individual NPIs as implemented in specific countries to inform national pandemic preparedness plans.

To better understand the evidence generated during the COVID-19 pandemic on the effectiveness of NPIs implemented in the UK, the UK Health Security Agency (UKHSA) first conducted a rapid mapping review.^11^^,^^12^ This identified and categorized the available evidence on NPIs used in the UK^11^^,^^12^; however, it did not critically appraise the included studies, nor synthesize the corresponding evidence. UKHSA then commissioned the current rapid review to update the original searches and critically appraise and synthesize the evidence. This work has two characteristics that distinguish it from other reviews of NPIs. Firstly, as explained above, it focuses specifically on NPIs as implemented in the UK. Secondly, whereas previous reviews included either interventional/observational studies or modelling studies, our review synthesizes evidence from all study designs, using a common methodological approach. By undertaking this review, our aim was to provide a thorough understanding of the evidence base for NPIs implemented in the UK during the COVID-19 pandemic that would inform UK policy and decision-making for pandemic preparedness and response.^13^

## Methods

We followed the PRISMA reporting guidelines.^14^ Three rapid review protocols, which grouped NPIs according to the categories defined by the mapping review, were registered on the Open Science Framework^15–17^; however, on completion of the rapid reviews, it was decided to combine them into a single publication.

### Search methods

The original search for the rapid mapping review was undertaken on 1 March 2023.^12^ An updated search using the same search strategy was conducted on 2 January 2024 to identify studies registered after 28 February 2023. We searched the following databases for published and unpublished work: Ovid MEDLINE (R) (to 29 December 2023), EMBASE (to 29 December 2023), National Institutes of Health (NIH) Covid Portfolio (to 2 January 2024) and Corona Central (to 2 January 2024). For search strategies, see supplementary materials.

The search strategy involved three concepts, separated by the Boolean operator AND: terms related to COVID-19, terms related to NPIs and terms related to the UK (using a validated UK geographic search filter).^18^^,^^19^

### Criteria for study inclusion

We included randomized controlled trials (RCTs), cohort, case–control, cross-sectional, quasi-experimental, natural experiments and modelling studies. Studies were included if they reported actual or simulated data (for modelling studies) on the UK general population or a sub-population of the UK, during the COVID-19 pandemic. We included studies that compared the NPI to either another NPI, the same NPI in another setting or no intervention. We also included within-person comparisons (before and after studies). Included outcomes were COVID-19 cases, hospitalization, mortality and reproduction number (*R*). We excluded studies based exclusively in health or social care settings, studies not published in English and studies evaluating travel and border measures (as these were not exclusively UK populations).

### Screening

The procedure for screening the initial search results is detailed in the rapid mapping review.^12^ As this was a rapid review, title and abstract screening for the updated search was undertaken in duplicate by two reviewers for a random 10% sample of the records identified, using the EPPI-Reviewer web version 4.^20^ The remaining 90% of records were screened by one reviewer with discussion with another reviewer in areas of uncertainty. Full-text screening was conducted by one reviewer and checked by a second, using EPPI-Reviewer. Studies eligible for the mapping review were rescreened to remove records that did not meet the narrower inclusion criteria for the rapid review.

### Data extraction and management

Two data extraction tools were developed and piloted for observational/interventional and modelling studies (see online repository https://osf.io/7czvs/). Data were extracted from each paper by a single reviewer and checked by a second reviewer, with disagreements resolved through discussion or the involvement of a third reviewer. We extracted data on the study purpose, study or model characteristics, study populations and COVID-19 outcomes.

### Assessment of methodological quality

We used the Quality Criteria Checklist for effectiveness studies^21^^,^^22^ to appraise the quality of observational and interventional studies because this tool can be used with a wide range of study designs. There is no validated tool to critically appraise modelling studies. We used a previously published tool with one modification to assess the modelling studies.^23^^,^^24^ Our approach, allowing reviewers to assess studies as having either ‘some’ or ‘no concerns’, was a pragmatic choice, given the time constraints of conducting a rapid review and the number of modelling studies (see supplementary materials). Quality assessment was done by a single reviewer, checked by a second with discrepancies resolved through discussion or by a third reviewer.

### Data analysis and synthesis

Based on the results of the rapid mapping review, it was decided that meta-analysis would be inappropriate because of the heterogeneity of study designs, variety of NPIs, format of outcome data, comparators and indices of effectiveness used by the studies. Results were therefore synthesized narratively. For each NPI, we made an overall assessment of the study findings with respect to the effectiveness of the NPI on COVID-19 outcomes. Findings of studies which reported a confidence interval (CI) were classified as suggestive of a protective effect, not suggestive or opposite to a protective effect (i.e. harmful). Where studies had reported an effect size without a CI, either numerically or, in the case of some modelling studies, graphically, findings were marked as unclear. Where our quality appraisal of modelling studies highlighted concerns about adequately accounting for uncertainty in the model itself, findings were marked as unclear, even if a CI was provided.

We synthesized the evidence by modifying the Synthesis without Meta-analysis (SWiM) guidelines,^25^ focusing on methodological quality, study relevance, consistency of results across studies and assessment of precision. Study design relevance was assessed by considering (i) how optimal the study designs were for assessing the effectiveness of the NPI and (ii) how heterogeneous the body of research was for that NPI in terms of study characteristics (e.g. study population, details of intervention, COVID-19 outcomes, comparators). Consistency of results was assessed by considering whether the reported direction of effect was the same across (i) interventional studies, (ii) observational studies and (iii) modelling studies. We considered whether precision of estimates had been assessed by recording the proportion of studies that provided a range of values around their numerical results.

We developed a decision algorithm for the assessment of certainty of evidence for each NPI category (see supplementary materials). We firstly assessed the direction of effect for the interventional/observational studies. If at least one study reported results in the opposite direction to the others, the effect of an NPI was considered to be inconclusive. If they were all in the same direction, modelling studies were then considered. If there were either no modelling studies or their results were in the same direction as the interventional/observational studies, we then considered the certainty of evidence based on quality appraisal, study heterogeneity and assessment of precision of all studies. If the results of the modelling studies were in a different direction to the interventional/observational studies, the certainty of evidence was downgraded one level. Levels of uncertainty ranged from very low to high.

## Results

### Search results

**Figure 1:**
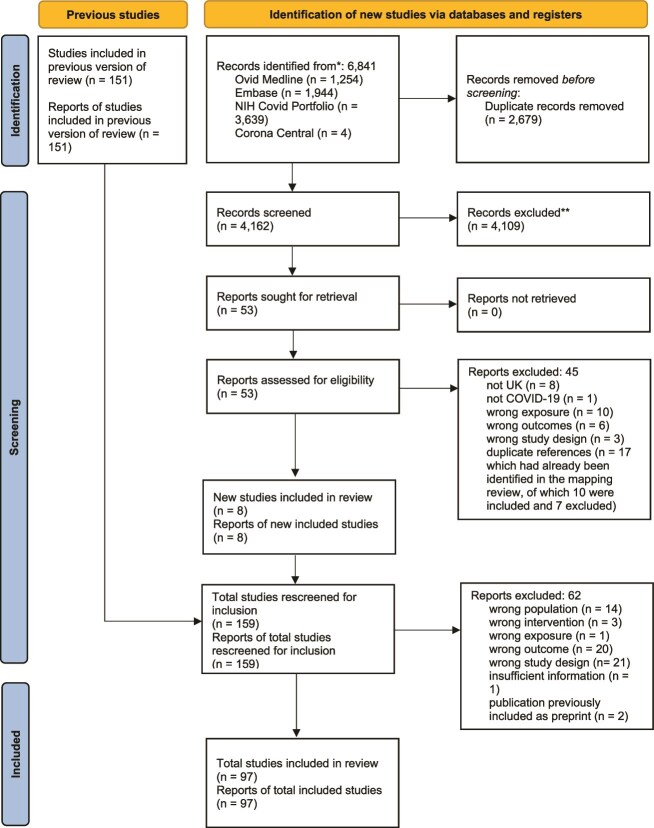
PRISMA flow diagram for study selection

A total of 151 studies were included in the UKHSA mapping review.^12^ The updated search yielded 6841 reports. After removal of duplicates, 4162 were screened and 53 were retrieved for full-text screening. Forty-five studies were excluded, and 8 studies were added to the initial 151 studies. These 159 studies were rescreened according to the narrower inclusion criteria of the present rapid review, resulting in the exclusion of 62 studies, leaving 97 studies for inclusion (see Fig. 1 for study selection, online repository https://osf.io/7czvs/ for excluded studies).

### Characteristics of included studies and NPIs

Table 2A in supplementary materials summarizes the characteristics of the 97 included studies, which reported data on the following 20 NPI categories: face covering giving protection to wearer^26–28^; face covering reducing risk of transmission^29–38^; physical distancing^26^^,^^27^^,^^29^^,^^39^; ventilation^28^^,^^33^^,^^35^; personal and household hygiene^26^^,^^27^^,^^33^; contact tracing^40–52^; National Health Service (NHS) contact tracing app^41^^,^^53^; asymptomatic testing^35^^,^^46^^,^^48^^,^^49^^,^^54–64^; isolation of cases^39^^,^^46^^,^^48^^,^^49^^,^^56^^,^^65–67^; isolation of contacts^30^^,^^66–69^; test and release of cases^70^^,^^71^; test and release of contacts^57^^,^^68^^,^^72–75^; NPIs that limit social contacts^42^^,^^76–83^; school-related NPIs^29^^,^^30^^,^^39^^,^^42^^,^^58^^,^^59^^,^^64^^,^^84–91^; work- and retail-related NPIs^37^^,^^52^^,^^63^^,^^92^; universal lockdown^89^^,^^93–113^; targeted and local lockdown^54^^,^^56^^,^^114^^,^^115^; tiered restrictions^84^^,^^116^; shielding^39^^,^^56^^,^^117–121^; and cohorting.^91^^,^^122^ Three of the included studies were interventional (two RCTs^72^^,^^73^ and one natural experiment^40^), nine were observational (two cohort,^118^^,^^120^ two case–control^74^^,^^117^ and five cross-sectional studies^26^^,^^27^^,^^29^^,^^93^^,^^119^), and the remaining 85 were modelling studies.

### Effectiveness of NPIs and certainty of the evidence

There was heterogeneity in the study designs and study characteristics (i.e. study settings and populations, details of how the NPI was implemented, COVID-19 outcomes reported, study comparators and format of results), and many modelling studies did not report numerical effect estimates with CIs. We summarized the findings of all studies narratively, reported the direction of effect (Table 1) and synthesized the evidence for each NPI category (Table 2).

**Table 1:** Table of findings by NPI category, ordered by study design and alphabetically.

NPI(s)	Study ID	Study design, details of NPI	Outcome(s)	Author’s conclusions	Quality appraisal*	Numerical results (95% confidence interval)	Overall assessment
Face covering use giving protection to the wearer	(Fairbanks *et al.*, 2023)^26^	Cross-sectional Type of face covering not specified	COVID-19 cases	Face covering use protects the wearer (Positive test result negatively correlated with face covering use, compared with not using a face covering)	Moderate	Not reported	Unclear
Face covering use giving protection to the wearer	(Francis *et al.*, 2023)^27^	Cross-sectional Type of face covering not specified	COVID-19 cases	Face covering use protects the wearer (Odds of infection significantly reduced with face covering use, compared with never using a face covering)	Moderate	OR = 0.19 (0.16, 0.23)	Suggestive
Face covering use giving protection to the wearer	(Miller *et al.*, 2022)^28^	Modelling Type of face covering not specified	Median dose of virus	Face covering use reduces dose of virus received by the wearer from an infected person.	Some concerns in 8 out of 10 categories	4.7× decrease in median dose of virus for 100% mask compliance, compared to 0%	Unclear
Face covering use reducing risk of transmission	(Marchant *et al.*, 2022)^29^	Cross-sectional Type of face covering not specified	COVID-19 cases	Face covering use does not reduce the risk of transmission. Use of face coverings by school staff (compared with not using face coverings) was not associated with lower odds of positive cases in primary schools	Moderate	OR = 2.1 (0.87, 5.05)	Not suggestive
Face covering use reducing risk of transmission	(Cuesta-Lazaro *et al.*, 2021)^30^	Modelling Type of face covering not specified	Cumulative COVID-19 deaths	Mask mandate in secondary schools (compared to no mask mandate in secondary schools) potentially reduces cumulative COVID-19 deaths. Combining mask mandate with physical distancing and better ventilation led to an estimated reduction in cumulative deaths of ~2500	Some concerns in 5 out of 10 categories	Combined impact of mask mandate, physical distancing and better ventilation in secondary schools potentially reduced cumulative COVID-19 deaths by ~2500 (95% CI presented graphically)	Suggestive
Face covering use reducing risk of transmission	(Donnat *et al.*, 2021)^31^	Modelling Type of face covering not specified	COVID-19 cases	Face covering use does not reduce the risk of transmission. At an indoor concert, more attendees wearing face coverings and shorter event duration reduced the number of infections but non-significantly.	Some concerns in 2 out of 10 categories	Mean (99% CI) infection rates for 100% vs. 0% mask compliance: 2.4 (0, 19) vs. 9.9 (0, 76)	Not suggestive
Face covering use reducing risk of transmission	(Fitz-Simon *et al.*, 2023)^32^	Modelling Type of face covering not specified	COVID-19 hospitalization	Mask mandates potentially reduce hospitalizations. A mask mandate implemented from early in the pandemic, with 90% population compliance, could potentially have significantly reduced hospitalizations in NI.	Some concerns in 5 out of 10 categories	Estimated 1089 (871, 1201) fewer hospitalizations under counterfactual scenario of early mask mandate, compared to actual number of observed hospitalizations.	Suggestive
Face covering use reducing risk of transmission	(Ghoroghi, Rezgui and Wallace, 2022)^33^	Modelling Surgical masks	COVID-19 cases	Wearing a surgical mask (compared to not wearing one) potentially reduces the probability of secondary cases in a university building, depending on level of compliance	Some concerns in 7 out of 10 categories	38–69% reduction in probability of secondary cases; however, there are some concerns with clarity on uncertainty of the model.	Unclear
Face covering use reducing risk of transmission	(Heald *et al.*, 2021)^34^	Modelling Type of face covering not specified	COVID-19 cases, COVID-19 hospitalization, COVID-19 mortality, *R* number	Mask mandates on public transport and in retail outlets potentially reduce transmission, hospitalization and mortality at population level. Effect magnitude is positively correlated with the *R* number.	Some concerns in 6 out of 10 categories	5%–17% reduction in cases if *R* = 0.8, 7%–25% reduction hospitalization if *R* = 1, 9%–31% reduction if *R* = 1.2; however, some concerns with clarity on uncertainty of the model.	Unclear
Face covering use reducing risk of transmission	(Moore *et al.*, 2021)^35^	Modelling Type of face covering not specified	COVID-19 cases	The most effective strategy for reducing transmission in post-secondary education settings was a combination of face covering use and good ventilation, followed by good ventilation alone, followed by face covering use alone	Some concerns in 5 out of 10 categories	Not reported	Unclear
Face covering use reducing risk of transmission	(Novakovic and Marshall, 2022)^36^	Modelling Type of face covering not specified	COVID-19 cases	Mask mandates potentially reduce case numbers. From 10 August to 1 October 2020, the estimated number of confirmed cases under the counterfactual scenario (no mask mandate) was almost 21 times the actual observed number of confirmed cases.	Some concerns in 2 out of 10 categories	Number of cases estimated by the model = 267 915 (258 861, 276 969) Number of observed cases = 12 792	Suggestive
Face covering use reducing risk of transmission	(Panovska-Griffiths *et al.*, 2021)^38^	Modelling Type of face covering not specified	COVID-19 cases	Mask mandates potentially reduce case numbers. Mask mandates in secondary schools in the autumn of 2020 would potentially have reduced but not prevented a second COVID-19 wave. Benefits were greater when effective mask coverage was high (30%).	Some concerns in 2 out of 10 categories	Not reported	Unclear
Face covering use reducing risk of transmission	(Ying and O’Clery, 2021)^37^	Modelling Type of face covering not specified	COVID-19 cases	Mask mandates in retail outlets potentially reduce case numbers. The number of infections and the chance of infection decreased with increased face covering use, particularly when combined with reduced occupancy levels.	Some concerns in 4 out of 10 categories	Combining mask mandate with halving customer arrival rates resulted in 96% fewer infections.	Unclear
Physical distancing	(Fairbanks *et al.*, 2023)^26^	Cross-sectional 2 m physical distancing	COVID-19 cases	2 m physical distance (compared to not maintaining 2 m physical distance) reduces the risk of positive COVID-19 test result.	Moderate	Not reported	Unclear
Physical distancing	(Francis *et al.*, 2023)^27^	Cross-sectional Being in a crowd of 10 or 100 people	COVID-19 cases	Being in a crowded indoor space with other people (compared to never being in a crowded indoor space) increases the risk of COVID-19 infection.	Moderate	OR = 1.62 (1.42, 1.85) (crowded space with 10 people) OR = 1.73 (1.53, 1.97) (crowded space with 100 people)	Suggestive
Physical distancing	(Marchant *et al.*, 2022)^29^	Cross-sectional School staff maintaining 2 m distance	COVID-19 cases	School staff maintaining 2 m distance from pupils or each other does not result in a reduced risk of COVID-19 cases occurring in primary schools.	Moderate	OR = 0.89 (0.33, 2.38) (2 m distance from pupils) OR = 2.85 (0.97, 8.37) (2 m distance from staff)	Not suggestive
Physical distancing	(Davies *et al.*, 2020)^39^	Modelling Physical distancing specified	COVID-19 cases, COVID-19 hospitalization, COVID-19 mortality, COVID-19 time to peak cases	Physical distancing is not associated with a significant reduction in COVID-19 case numbers, peak demand for non-ICU hospital beds, mortality or time to peak cases. Data are for physical distancing vs. no physical distancing (95% CI).	Some concerns in 4 out of 10 categories	Case numbers (millions): 16 (6.2, 24) vs. 23 (13, 30) Peak non-ICU beds (thousands): 190 (39, 390) vs. 390 (110, 700) Deaths (thousands): 230 (81, 370) vs. 350 (170, 480) Time to peak cases (weeks): 19 (12, 37) vs. 12 (9, 20)	Not suggestive
Ventilation	(Ghoroghi, Rezgui and Wallace, 2022)^33^	Modelling Natural (windows), mechanical (supply and extraction) and mixed ventilation	COVID-19 cases	The most effective at preventing transmission was mixed mechanical and natural ventilation enhanced with fans; however, ventilation alone was insufficient to prevent transmission of the Delta variant.	Some concerns in 7 out of 10 categories	Not reported	Unclear
Ventilation	(Miller *et al.*, 2022)^28^	Modelling No details on ventilation specified	Airborne dose of SARS-CoV-2	Inverse association between ventilation rate and mean and median airborne dose received; however, the reduction did not scale with the inverse of the ventilation rate alone.	Some concerns in 8 out of 10 categories	Not reported	Unclear
Ventilation	(Moore *et al.*, 2021)^35^	Modelling Use of air conditioning and fans	COVID-19 cases	The most effective strategy for reducing transmission was a combination of face coverings and good ventilation, followed by good ventilation alone, followed by face coverings alone.	Some concerns in 5 out of 10 categories	Not reported	Unclear
Personal and household hygiene	(Fairbanks *et al.*, 2023)^26^	Cross-sectional Hand washing or sanitizing before and/or after an activity	COVID-19 cases	Positive COVID-19 test results were negatively correlated with hand hygiene	Moderate	Not reported	Unclear
Personal and household hygiene	(Francis *et al.*, 2023)^27^	Cross-sectional Hand hygiene on arrival home or before eating, cleaning surfaces, touching face (any frequency compared with never)	COVID-19 cases	Hand hygiene on arrival home significantly reduced the odds of infection. Hand hygiene before eating significantly increased the odds of infection. This may be the result of recall bias. Cleaning surfaces such as doors and taps significantly increased the odds of infection. This may be the result of recall bias. There was no evidence of an association between avoiding touching one’s face and COVID-19 infection.	Moderate	Handwashing on arrival home OR = 0.63 (0.48, 0.83)	Suggestive
						Handing washing before eating OR = 1.49 (1.14, 1.94) Cleaning surfaces OR = 1.38 (1.15, 1.64)	Opposite to expected
						Touching face OR = 1.17 (0.99, 1.38)	Not suggestive
Personal and household hygiene	(Ghoroghi, Rezgui and Wallace, 2022)^33^	Modelling Face covering and hand hygiene	COVID-19 cases	Ventilation alone cannot be relied on to prevent transmission of the Delta variant. This must be combined with the use of face coverings and hand hygiene.	Some concerns in 7 out of 10 categories	Not reported	Unclear
Contact tracing	(Findlater *et al.*, 2022)^40^	Natural experiment Failure of contact tracing system	Risk of infection (primary or secondary contacts), hospital admissions, mortality	No evidence of a difference in secondary attack rates (SARs) overall, nor of a difference in hospitalizations or mortality among primary or secondary contacts in the delay or control group.	High	Difference in SAR [all primary contacts]: 0.1% (−0.4, 0.2) OR hospital admission [primary contacts]: 1.1 (1.0, 1.2) OR 28-day mortality [primary contacts]: 0.8 (0.4, 1.6)	Not suggestive
Contact tracing	(Almagor and Picascia, 2020)^41^	Modelling Smartphone-based contact tracing	COVID-19 cases	Smartphone-based contact tracing is a viable epidemic mitigation strategy; as larger fractions of society adopt the contact tracing app, the spread of the virus is increasingly reduced.	Some concerns in 5 out of 10 categories	Not reported	Unclear
Contact tracing	(Brooks-Pollock *et al.*, 2021)^42^	Modelling Contact tracing details not specified	Secondary infections per case Reproduction number	Findings support the use of contact tracing as a key part of epidemic control but needs to be highly effective: tracing 20% of contacts is insufficient to prevent epidemic growth if schools are fully open.	Some concerns in 6 out of 10 categories	Not reported	Unclear
Contact tracing	(Davis *et al.*, 2021)^43^	Modelling Contact tracing details not specified	Reproduction number	Well-implemented contact tracing could bring small but potentially important benefits to controlling and preventing outbreaks, providing up to a 15% reduction in R.	Some concerns in 5 out of 10 categories	Not reported	Unclear
Contact tracing	(Endo *et al.*, 2021)^44^	Modelling Contact tracing details not specified	Secondary infections per case	Backward contact tracing [in addition to forward tracing] has the potential to identify a large proportion of infections because of the observed over-dispersion in COVID-19 transmission.	Some concerns in 5 out of 10 categories	Not reported	Unclear
Contact tracing	(Fyles *et al.*, 2021)^45^	Modelling Contact tracing details not specified	COVID-19 cases	Implementing a contact tracing, isolation and quarantine policy could contribute to controlling the SARS-CoV-2 epidemic if lockdown levels are partially relaxed but not if relaxed completely.	Some concerns in 2 out of 10 categories	For each 1 day increase in mean testing delay, the epidemic growth rate was associated with an increase of 0.0138 (0.009, 0.018)	Suggestive
Contact tracing	(Grassly *et al.*, 2020)^46^	Modelling Contact tracing details not specified	Reproduction number	Test and trace can further reduce *R*, in addition to results achieved by self-isolation, but this is dependent on the proportion of cases and contacts identified, and the timeliness of follow-up.	Some concerns in 5 out of 10 categories	Test and trace can reduce R by 26% (14,35) on top of reductions achieved by self-isolation, if 80% of cases and contacts are identified	Suggestive
Contact tracing	(He *et al.*, 2021)^47^	Modelling Contact tracing details not specified	Reproduction number	Test, trace and isolate (TTI) strategies have a moderate effect on *R* and need to be implemented together with other NPIs.	Some concerns in 4 out of 10 categories	For scenario 1 (no social restrictions; symptomatic households quarantined): With no TTI, *R* = 2.34 ± 0.06 With symptom-based TTI, *R* = 1.94 ± 0.05 With test-based TTI, *R* = 2.02 ± 0.05 With test-based TTI with contact tracing, *R* = 2.04 ± 0.05	Suggestive
Contact tracing	(Hill *et al.*, 2021a)^52^	Modelling Contact tracing details not specified	COVID-19 cases	Increased adherence to test-and-trace measures can significantly reduce the size of an outbreak	Some concerns in 5 out of 10 categories	Isolation, test & trace: With an increase from 0% adherence to 100% adherence, the overall outbreak size was reduced by 50%, and the peak prevalence was reduced by 75%.	Unclear
Contact tracing	(Hill *et al.*, 2021b)^48^	Modelling Contact tracing details not specified	COVID-19 cases	Effective contact tracing curbed transmission of SARS-CoV-2 in a student population, if broadly adhered to.	Some concerns in 4 out of 10 categories	If everyone adhered to TTI measures, 22% (7,41) of the student population could be infected during the autumn term, vs 69% (56,76) if no one adhered.	Suggestive
Contact tracing	(Kucharski *et al.*, 2020)^49^	Modelling Contact tracing details not specified	COVID-19 cases	Strategies that combine isolation of symptomatic cases with testing and quarantine of their contacts reduce the effective R number more than mass testing or self-isolation alone.	Some concerns in 6 out of 10 categories	Reduction in effective R number due to: Self-isolation and household quarantine Manual tracing of all contacts: 64% Manual tracing of acquaintances only: 57% App-based contact tracing only: 47%	Unclear
Contact tracing	(Lucas *et al.*, 2021)^50^	Modelling Contact tracing details not specified	COVID-19 cases	Within contact tracing strategies, policies that increase self-isolation rates at the expense of self-report rates are unlikely to improve the effectiveness of contact tracing.	Some concerns in 5 out of 10 categories	Not reported	Unclear
Contact tracing	(Stocks *et al.*, 2023)^51^	Modelling Contact tracing details not specified	COVID-19 cases	The spread of COVID-19 from symptomatic index cases is greatly reduced with contact tracing, but the reduction for asymptomatic cases is minimal.	Some concerns in 6 out of 10 categories	Contact tracing can reduce transmission from symptomatic index cases by up to 56% but by <3% for asymptomatic cases.	Unclear
NHS COVID-19 contact tracing app	(Almagor and Picascia, 2020)^41^	Modelling Smartphone-based contact-tracing app	COVID-19 cases	Smartphone-based contact tracing is a viable epidemic mitigation strategy; as larger fractions of society adopt the contact tracing app, the spread of the virus is increasingly reduced.	Some concerns in 5 out of 10 categories	Not reported	Unclear
NHS COVID-19 app (different lengths of notification window)	(Leng *et al.*, 2022b)^53^	Modelling NHS COVID-19 app as used in England and Wales	Reproduction number	The reduction in R from app use (compared to non-use) increases with notification window length, but there are limited further benefits for notification windows longer than five days.	Some concerns in 7 out of 10 categories	Not reported	Unclear
Asymptomatic testing	(Alsing, Usher and Crowley, 2020)^54^	Modelling Asymptomatic testing details not specified	COVID-19 cases	Where an appreciable proportion of transmission occurs within, rather than between, communities, then targeted community-level interventions (including mass testing) can be effective at containing SARS-CoV-2 outbreaks.	Some concerns in 7 out of 10 categories	Not reported	Unclear
Asymptomatic testing	(Drakesmith *et al.*, 2022)^55^	Modelling Asymptomatic testing details not specified	COVID-19 cases Hospitalizations ICU admissions Deaths	As a result of the whole-area testing pilot, a non-negligible number of cases, hospitalizations and deaths, which would otherwise have occurred, were likely to have been prevented.	Some concerns in 4 out of 10 categories	A conservative estimate of 360 (311,418) cases were prevented by the mass testing. An estimated 24 (16,36) hospitalizations, 5 (3,6) ICU admissions and 15 (11,20) deaths were prevented.	Suggestive
Asymptomatic testing	(Goscé *et al.*, 2020)^56^	Modelling Asymptomatic testing details not specified	COVID-19 cases Deaths	A strategy that combines continued lockdown with universal testing with case isolation, contact tracing and isolation and facemask use by the general population is the only scenario with the potential for higher effectiveness in reducing infections, deaths and lockdown duration, compared to ongoing lockdown with no additional interventions.	Some concerns in 5 out of 10 categories	Universal testing after lockdown is lifted could lead to a reduction of 40% in the peak of infections and 12% in the peak cumulative deaths, compared to lifting lockdown with no testing.	Unclear
Asymptomatic testing	(Grassly *et al.*, 2020)^46^	Modelling Asymptomatic testing details not specified	Reproduction number, *R*	Weekly screening of healthcare workers would reduce their contribution to SARS-CoV-2 transmission by around one quarter, on top of reductions achieved by self-isolation for symptomatic cases.	Some concerns in 5 out of 10 categories	Weekly screening of healthcare workers and a 24-h delay from testing to self-isolation would reduce their contribution to SARS-CoV-2 transmission (*R*) by 23% (16,40)	Suggestive
Asymptomatic testing	(Hill *et al.*, 2021b)^48^	Modelling Asymptomatic testing in university students	COVID-19 cases	Mass testing had the ability to significantly reduce overall infection levels, if performed regularly and adhered to.	Some concerns in 4 out of 10 categories	Adherence to test, trace and isolation 22% (7,41) of student population could be infection compared to 69% (56,76) with zero adherence.	Suggestive
Asymptomatic testing	(Kucharski *et al.*, 2020)^49^	Modelling Asymptomatic testing details not specified	COVID-19 cases	Strategies that combine isolation of symptomatic cases with testing and quarantine of their contacts reduce the effective *R* number more than mass testing or self-isolation alone.	Some concerns in 6 out of 10 categories	Mass testing of 5% of the population per week corresponded to a 2% mean reduction in effective *R* number	Unclear
Asymptomatic testing	(Kunzmann *et al.*, 2021)^57^	Modelling Asymptomatic testing details not specified	COVID-19 cases	Containment depends on the fraction of asymptomatic cases, and policies incorporating regular asymptomatic screening tests are more robust.	Some concerns in 2 out of 10 categories	No numerical results but results reported in figures with confidence intervals	Suggestive
Asymptomatic testing	(Leng *et al.*, 2022c)^59^	Modelling Asymptomatic testing details not specified	COVID-19 cases	Twice-weekly mass testing can result in lower levels of infections than a strategy of isolating year-group bubbles, particularly if combined with serial contact testing.	Some concerns in 6 out of 10 categories	No numerical results but results reported in figures with confidence intervals	Suggestive
Asymptomatic testing	(Leng *et al.*, 2022a)^58^	Modelling Asymptomatic testing details not specified	COVID-19 cases	Results support the importance of mass testing via lateral flow tests in reducing transmission, despite the lower sensitivity of lateral flow tests compared to PCR tests.	Some concerns in 4 out of 10 categories	No numerical results but results reported in figures with confidence intervals	Suggestive
Asymptomatic testing	(Moore *et al.*, 2021)^35^	Modelling Asymptomatic testing details not specified	COVID-19 cases	Testing can help to reduce the total number of infections but cannot ever significantly mitigate infection spread.	Some concerns in 5 out of 10 categories	No reported	Unclear
Asymptomatic testing	(Sandmann *et al.*, 2020)^60^	Modelling Asymptomatic testing details not specified	COVID-19 cases	Testing all workers not only reduces the risk of workplace transmission the most but also increases staff absence and required testing capacity. Testing workers in quarantine reduces absence by releasing those who test negative but increases the risk of workplace transmission.	Some concerns in 9 out of 10 categories	No reported	Unclear
Asymptomatic testing	(Silva *et al.*, 2023)^61^	Modelling Asymptomatic testing details not specified	COVID-19 cases	Asymptomatic testing with lateral flow tests is particularly effective when the growth rate corresponds to a weekly doubling in the number of cases. Regular asymptomatic testing with lateral flow tests can be a viable alternative to national lockdowns.	Some concerns in 3 out of 10 categories	Estimated decrease in COVID-19 infections, based on testing once per week:–100% adherence (POLYMOD): 3.9%–32.2%—100% adherence (CoMix): 3.9%–32.9% (POLYMOD and CoMix are two large surveys of contact patterns. POLYMOD took place across Europe in 2008 and was used to represent pre-COVID-19 patterns, while CoMix took place in the UK during the pandemic.)	Suggestive
Asymptomatic testing	(Warne *et al.*, 2021)^62^	Modelling Asymptomatic testing details not specified	Reproduction number, *R*	Study provides evidence for the efficacy of regular asymptomatic screening, through enhanced case ascertainment, pre-emptive quarantine and reduction in risk of symptomatic COVID-19 following a negative test result.	Some concerns in 6 out of 10 categories	Weekly screening of all students (as implemented during weeks 8–9 of the programme) resulted in a 31% reduction in R0 from a median of 1.78 (1.37,2.23) to a median of 1.22 (0.82,1.62)	Suggestive
Asymptomatic testing	(Whitfield *et al.*, 2023)^63^	Modelling Asymptomatic testing details not specified	COVID-19 cases	Combining testing measures with NPIs such as social distancing, work from home and masking can reduce risk of infection in the workplace and reduce the costs of employee isolation.	Some concerns in 4 out of 10 categories	Not reported	Unclear
Asymptomatic testing	(Woodhouse *et al.*, 2022)^64^	Modelling Asymptomatic testing details not specified	COVID-19 cases	A regular rapid lateral flow test testing regime has significant benefits in reducing transmission and is more effective than bubble or class exclusion.	Some concerns in 4 out of 10 categories	Not reported	Unclear
Isolation of cases	(Davies *et al.*, 2020)^39^	Modelling Self-isolation of symptomatic people	COVID-19 cases Deaths	Self-isolation alone could not reduce R0 enough to bring about a sustained decline in the incidence of new infections but was more effective in combination with other NPIs.	Some concerns in 4 out of 10 categories	Total COVID-19 cases Base scenario: 23 m (13–30 m) Self-isolation: 17 m (6.1–25 m) Combination: 17 m (6.5–26 m) Total COVID-19 deaths Base scenario: 350 000 (170 000–480 000) Self-isolation: 240 000 (78 000–400 000) Combination: 260 000 (85 000–410 000)	Suggestive
Isolation of cases	(Farkas and Chatzopoulos, 2021)^65^	Modelling Self-isolation of symptomatic people	COVID-19 cases	Self-quarantine has a significant impact at the start of an outbreak, due to delaying the onset of the peak, but has a negligible impact on peak case numbers.	Some concerns in 6 out of 10 categories	Not reported	Unclear
Isolation of cases	(Goscé *et al.*, 2020)^56^	Modelling Self-isolation of positive cases	COVID-19 cases Deaths	A strategy that combines continued lockdown with universal testing with case isolation, contact tracing and isolation and facemask use by the general population is the only scenario with the potential for higher effectiveness in reducing infections, deaths and lockdown duration, compared to ongoing lockdown with no additional interventions.	Some concerns in 5 out of 10 categories	If lockdown is lifted but symptomatic people continue to self-isolate, there will be an estimated 1.8 million cases and 263 000 cumulative deaths on the day of the peak.	Unclear
Isolation of cases	(Grassly *et al.*, 2020)^46^	Modelling Self-isolation of all symptomatic people, or of symptomatic people with positive PCR tests	Reproduction number, *R*	Self-isolation following onset of symptoms results in a reduction in COVID-19 transmission in the community, although this depends on the proportion of asymptomatic and presymptomatic infections.	Some concerns in 5 out of 10 categories	A reduction in R of 47% (32,55) is observed if all symptomatic individuals self-isolated, assuming self-isolation was 100% effective in preventing transmission.	Suggestive
Isolation of cases	(Hill *et al.*, 2021b)^48^	Modelling Room-based isolation of symptomatic students (until end of symptoms)	COVID-19 cases	Findings demonstrate the efficacy of isolation and tracing measures in controlling the spread of SARS-CoV-2, if broadly adhered to.	Some concerns in 4 out of 10 categories	If everyone adhered to test, trace & isolation measures, 22% (7,41) of the student population could be infected during the autumn term, vs 69% (56,76) if no one adhered.	Suggestive
Isolation of cases	(Kucharski *et al.*, 2020)^49^	Modelling Self-isolation of symptomatic people at home or outside the home, with or without whole-household quarantine	COVID-19 cases	Strategies that combine isolation of symptomatic cases with testing and quarantine of their contacts reduce the Reff more than mass testing or self-isolation alone.	Some concerns in 6 out of 10 categories	Mean reductions in COVID-19 transmission: Self-isolation of symptomatic cases at home: 29% Self-isolation of symptomatic cases outside the home: 35% Self-isolation plus household quarantine: 37%	Unclear
Isolation of cases	(Nadim, Ghosh and Chattopadhyay, 2021)^66^	Modelling Self-isolation of symptomatic people	COVID-19 cases	The effectiveness of isolation of contacts or cases depends on the relative infectiousness of contacts/cases. Both measures appear to be effective in reducing COVID-19 transmission in the UK.	Some concerns in 4 out of 10 categories	Not reported	Unclear
Isolation of cases	(Wells *et al.*, 2020)^67^	Modelling Self-isolation of symptomatic people	COVID-19 cases	Trace and isolation of symptomatic individuals was of limited efficacy in lowering epidemic size, unless overall transmission rate is kept relatively low.	Some concerns in 4 out of 10 categories	Not reported	Unclear
Isolation of contacts	(Cuesta-Lazaro *et al.*, 2021)^30^	Modelling Class quarantine—all class-based contacts isolate for 10 days	COVID-19 deaths	Classroom quarantines were found to be effective at reducing the growth of the pandemic, but the best results were achieved by reducing contact intensity.	Some concerns in 5 out of 10 categories	Class quarantine results in lower cumulative number of deaths (∼1500 fewer deaths). Confidence intervals provided in graphical outputs.	Suggestive
Isolation of contacts	(Nadim, Ghosh and Chattopadhyay, 2021)^66^	Modelling Self-isolation of contacts	COVID-19 cases	The effectiveness of isolation of contacts or cases depends on the relative infectiousness of contacts/cases. Both measures appear to be effective in reducing COVID-19 transmission in the UK.	Some concerns in 4 out of 10 categories	Not reported	Unclear
Isolation of contacts	(Quilty *et al.*, 2021)^68^	Modelling Self-isolation of contacts for 14 days	COVID-19 cases	14 days of quarantine after last exposure can reduce onward transmission from secondary cases. The effectiveness of contact tracing can be limited by low adherence to quarantine.	Some concerns in 8 out of 10 categories	14-day quarantine can prevent 48% (18,79) of onward transmission; however, there are some concerns with clarity on uncertainty of the model.	Unclear
Isolation of contacts	(Wells *et al.*, 2020)^67^	Modelling Described as isolation of non-symptomatic individuals identified by test-and-trace	COVID-19 cases	Isolation of non-symptomatic infected individuals is pivotal to reducing overall epidemic size over a wider range of transmission scenarios	Some concerns in 4 out of 10 categories	Not reported	Unclear
Isolation of contacts	(Zhang *et al.*, 2022)^69^	Modelling Self-isolation of contacts	COVID-19 cases	Variation in self-isolation and quarantine rates can considerably affect the duration of outbreaks, attack rates and peak timing.	Some concerns in 6 out of 10 categories	Not reported	Unclear
Test and release strategies—cases	(Bays *et al.*, 2022)^70^	Modelling Isolation for up to 5 days plus daily LFD testing, up to a total of 10 days	Infectious cases released from isolation	Use of LFD testing alongside isolation can deliver a reduction in the risk of releasing individuals who are still infectious while simultaneously decreasing the average time spent in isolation.	Some concerns in 8 out of 10 categories	The fixed 5-day isolation period approach to release on day 5 with one, two or three negative LFD results provides a 46.5%, 74.0% and 81.4% decrease in infectious releases, respectively.	Unclear
Test and release strategies—cases	(Quilty, Pulliam and Pearson, 2022)^71^	Modelling 3, 5 or 7 days of isolation plus 1, 2 or 3 continuous daily negative lateral flow tests required for release.	Infectious cases released from isolation	The number of infectious days in the community can be reduced to almost zero by requiring at least two consecutive days of negative tests, after initially testing positive.	Some concerns in 10 categories	Not reported	Unclear
Test and release strategies – contacts	(Love *et al.*, 2022a)^72^	Randomised controlled trial Daily testing using LFDs for 7 days, no isolation if negative. PCR tests on day 1 and on positive LFD test or last day of testing.	COVID-19 cases	Daily contact testing with 24-h exemption from self-isolation for essential activities appears to be comparable to self-isolation.	High	Difference in secondary attack rate (overall): −1.20% (−2.30, −0.20)	Suggestive
Test and release strategies—contacts	(Young *et al.*, 2021)^73^	Randomised controlled trial Daily LFD testing for 7 days; continued school attendance if test negative.	COVID-19 cases	Daily contact testing of school-based contacts was comparable to self-isolation for control of COVID-19 transmission, with similar rates of symptomatic infections among students and staff with both approaches.	High	COVID-19 infections (PCR-confirmed) during *N* days at risk [intention-to-treat adjusted incidence risk ratio (aIRR)]: 0.96 (0.75, 1.22)	Suggestive
Test and release strategies—contacts	(Love *et al.*, 2022b)^74^	Case–control study Daily testing using LFDs for 7 days, no isolation if negative. PCR test if positive LFD test or on last day of testing.	COVID-19 cases	Daily testing using LFDs was acceptable to contacts of cases, and there was likely to be public health benefit in routinely offering tests to contacts to increase case ascertainment.	Moderate	Difference in secondary attack rates: 6.3% (95% CI 3.4%–11.1%) in the study group and 7.6% (95% CI 7.3%–7.8%) in the control group—no significant difference.	Not suggestive
Test and release strategies—contacts	(Ferretti *et al.*, 2021)^75^	Modelling Daily LFD testing for 7 days, starting 3 days after exposure. No isolation if negative.	COVID-19 cases	Assuming intermediate adherence in both cases, the two strategies (daily contact testing vs. quarantine of traced contacts) reduce onward transmission by a similar amount, and the social/economic costs for daily contact testing were much lower.	Some concerns in 5 out of 10 categories	Not reported	Unclear
Test and release strategies—contacts	(Kunzmann *et al.*, 2021)^57^	Modelling Daily LFD testing of school-based contacts for up to 7 school days, no isolation if negative.	COVID-19 cases	Test for release needs a symptomatic index case to trigger dynamic testing (as opposed to asymptomatic screening), so struggles to contain outbreaks.	Some concerns in 2 out of 10 categories	No numerical results but reported in figures with confidence intervals	Not suggestive
Test and release strategies—contacts	(Quilty *et al.*, 2021)^68^	Modelling Varying test-and-release strategies (7 day isolation + negative tests, or 5 days of continuous negative tests)	COVID-19 cases	A lateral flow antigen (LFA) test 7 days after exposure, with quarantine from tracing until testing, or alternatively daily testing with LFA tests for 5 days after tracing, might avert a similar proportion of COVID-19 cases to that of 14-day quarantine.	Some concerns in 8 out of 10 categories	Reduction in onward transmission [LFA test after 7 days] RR: 1.00 (0.82, 1.28) Reduction in onward transmission [daily testing for 5 days] RR: 1.04 (0.69, 1.79); however, there are some concerns with clarity on uncertainty of the model.	Unclear
Group of NPIs that limit social contacts (Scale UK)	(Brooks-Pollock *et al.*, 2021)^42^	Modelling Multiple NPI such as physical distancing, face coverings, contact tracing and school closures to reduce contact	R number	Reduction in R number for reduction in work and leisure contacts	Some concerns in 6 out of 10 categories	Opening primary and secondary school *R* = 1.22 (1.02,1.53). Opening primary schools alone *R* = 0.89 (0.82,0.97)	Suggestive
Group of NPIs that limit social contacts (Scale UK)	(Hill, 2023)^76^	Modelling Christmas bubble scenarios with household mixing	Number and percentage increase in cumulative infections	All scenarios that allowed mixing beyond external household led to increase in infections	Some concerns in 5 out of 10 categories	Cumulative infections across all ages for exclusive bubbles 1.05% (0.95,1.15), non-exclusive fixed bubbles 1.05% (0.95,1.15).	Not suggestive
Group of NPIs that limit social contacts (Scale UK)	(Lovell-Read, Shen and Thompson, 2022)^77^	Modelling Combination of lockdown, school closures, social distancing and surveillance.	Probability of local outbreaks	Reducing contacts outside of school and workplaces was the most effective intervention. Mixed interventions were more effective than individual interventions	Some concerns in 9 out of 10 categories	Not reported	Unclear
Group of NPIs that limit social contacts (Scale England)	(Hilton *et al.*, 2022)^78^	Modelling Impact of household mixing, temporary relaxation of NPI and out of household isolation	Transmission rate	Larger temporary household bubbles and longer mixing periods were associated with higher prevalence	Some concerns in 6 out of 10 categories	Not reported	Unclear
Group of NPIs that limit social contacts (Scale England)	(Leng *et al.*, 2021)^79^	Modelling Contact clustering and social bubbles	Transmission rate and mortality	Two households can allow increased social contact whilst limiting additional risk; social bubbles reduce number of infections	Some concerns in 7 out of 10 categories	Social bubbles reduced fatalities by 42%	Unclear
Group of NPIs that limit social contacts (Scale England)	(Sonabend *et al.*, 2021)^80^	Modelling Examined the impact of four steps of the roadmap out of lockdown	COVID-19 cases, hospital admissions and deaths	Delaying step 4 until 19 July reduced all outcome measures.	Some concerns in 2 out of 10 categories	No numerical results but results reported in figures with confidence intervals	Suggestive
Group of NPIs that limit social contacts (Scale England)	(Ziauddeen, Subramaniam and Gurdasani, 2021)^81^	Modelling Easing of lockdown measures	Number of excess cases and deaths	Reported the excess in cases and deaths due to easing lockdown.	Some concerns in 7 out of 10 categories	Number of excess cases 257 (108 492), number of excess deaths 26 447 (11 105,50 549); however, there are some concerns with clarity on uncertainty of the model.	Unclear
Group of NPIs that limit social contacts (Scale Hebridean islands in Scotland)	(Ruget *et al.*, 2021)^82^	Modelling Limitation in social contacts and restriction of movement to mainland	Number of infections	Results suggested that it was more effective to limit contacts than to reduce movement to and from the mainland.	Some concerns in 5 out of 10 categories	Not reported	Unclear
Group of NPIs that limit social contacts (Scale Northeast London)	(Cheetham *et al.*, 2021)^83^	Modelling Limitations in social contacts	COVID-19 cases, deaths and hospitalizations related to number of contacts	Increase in daily contacts >6 led to more cases/deaths and hospitalizations	Some concerns in 7 out of 10 categories	Not reported	Unclear
Group of school related NPIs (School intervention)	(Marchant *et al.*, 2022)^29^	Cross-sectional Teaching indoors and outdoors, availability of clubs.	Odds ratio (OR) of COVID-19 cases	No association was found for the following interventions and COVID-19 cases: class mixing, indoor and outdoor teaching and use of clubs	Moderate	OR for positive case for class mix 1.06 (0.53,2.13), for breakfast club OR 0.67 (0.28,1.64), clubs 1.99 (0.85,4.71)	Not suggestive
Group of school-related NPIs (School closures)	(Davies *et al.*, 2020)^39^	Modelling School alone and in combination with other NPIs (physical distancing, self-isolation, shielding)	Number of cases and deaths, ICU beds and non-ICU beds in peak weeks	When used alone, school closure was not able to decrease healthcare needs to below capacity.	Some concerns in 4 out of 10 categories	In peak week, cases reduced to 2.7 million (420 000–6 million) from 3.9 million (1.3–6.9 million). Death reduced to 39 000 (5.7 000–86 000) from 57 000 (17 000–100 000).	Suggestive
Group of school-related NPIs (School closures)	(Davies *et al.*, 2021)^84^	Modelling School closures with circuit breaker/fire breaker lockdowns	Transmission, hospital admissions, deaths	Reduction seen with school closures during circuit or fire breaker lockdowns	Some concerns in 2 out of 10 categories	Reduction of COVID-19 transmission with school closures and circuit breaker by 35% (30,41) in Northern Ireland, 44% (37,49) in Wales and 36% (29,42) in England.	Suggestive
Group of school-related NPIs (Return to school)	(Aspinall *et al.*, 2020)^85^	Modelling Return to school in cohorts of children and teachers	Number of infected persons, number of schools with one or more infected person	The relative number of schools with infected persons increases with number of returning children and teachers	Some concerns in 3 out of 10 categories	If one third of children returned to school between 178 and 924 schools (1%–5% of schools) would have an infected person. Values and CI provided for each scenario.	Suggestive
Group of school-related NPIs (Return to school)	(Brooks-Pollock *et al.*, 2021)^42^	Modelling Return to school opening primary and secondary schools	*R* number	Opening both primary and secondary schools has larger impact on *R* than primary schools alone	Some concerns in 6 out of 10 categories	*R* number 0.80 (0.82,0.97) with primary schools opening increase to *R* 1.22 (1.02,1.53) with both primary and secondary school opening.	Suggestive
Group of school-related NPIs (Return to school)	(Keeling *et al.*, 2021b)^86^	Modelling Return to school re-opening with variation in class size and year returning to school	Secondary infections, clinical cases, *R* number	More year groups returning the bigger the impact with secondary school classes having a greater effect than primary school classes,	Some concerns in 4 out of 10 categories	No numerical results but results reported in figures with confidence intervals	Suggestive
Group of school related NPIs (Return to school)	(Munday *et al.*, 2021a)^88^	Modelling Return to school with partial or full reopening	*R* number	Full school reopening caused a higher rise in R compared to partial	Some concerns in 4 out of 10 categories	Full school opening increased R by a factor of 1.3,1.9 and partial reopening increased *R* by smaller amount (by factor of 0.9,1.2).	Suggestive
Group of school-related NPIs (Return to school)	(Munday *et al.*, 2021b)^87^	Modelling Return to school with reopening allowing different year groups	Transmission between schools and pupil households	Opening a small selection of years only presents a small risk, but, when secondary schools years are included, there is a higher risk.	Some concerns in 5 out of 10 categories	No numerical results but reported in figures with confidence intervals	Suggestive
Group of school-related NPIs (Return to school)	(Panovska-Griffiths *et al.*, 2020)^90^	Modelling Partial and full reopening of school with testing and contact tracing	Daily cumulative number of infection and deaths, *R* number	Reopening schools full- or part-time from September 2020 alongside other relaxations would induce a second wave.	Some concerns in 6 out of 10 categories	Not reported	Unclear
Group of school-related NPIs (Return to school)	(Panovska-Griffiths *et al.*, 2022)^89^	Modelling Partial and full reopening of schools with full and partial lockdown	Daily cumulative number of infection and deaths, *R* number	Once lockdown relaxed with some form of school reopening *R*, there would be increase in cases and *R* number	Some concerns in 2 out of 10 categories	Not reported	Unclear
Group of school-related NPIs (School intervention)	(Cuesta-Lazaro *et al.*, 2021)^30^	Modelling Class quarantine and variation in contacts within school using face coverings, isolation and social distancing	Percentage of infections and deaths	Reducing interaction intensity (class quarantine and face coverings) can reduce cumulative deaths.	Some concern in 5 out of 10 categories	No numerical results but results reported in figures with confidence intervals	Suggestive
Group of school-related NPIs (School intervention)	(Kaiser, Kretschmer and Leszczensky, 2021)^91^	Modelling Effects of cohorting compared to no cohorting within school environment	Proportion of outbreaks, proportion of infected students, proportion quarantined	Can reduce incidence within classroom with network-based strategies that factor in out of school contacts stopping superspreading	Some concerns in 7 out of 10 categories	Not reported	Unclear
Group of school-related NPIs (School intervention)	(Leng *et al.*, 2022c)^59^	Modelling School bubbles, mass testing, serial contact testing	Testing rate and number of absences from school	Twice weekly mass testing was more effective than school bubbles.	Some concerns in 6 out of 10 categories	44% (9,118) increase in pupil-to-pupil transmission due to falling adherence to within school measures	Suggestive
Group of school-related NPIs (School intervention)	(Leng *et al.*, 2022a)^58^	Modelling School bubbles with various testing strategies	Transmission, infection rate, school absence	Compared to isolating year group bubbles, a strategy of twice weekly mass testing (without further control measures) was more effective at reducing transmission	Some concerns in 4 out of 10 categories	Without control measures, a mean of 16.6% (6.8,42.9), of all pupils had been infected by the end of the half-term; however, there are some concerns with clarity on uncertainty of the model.	Unclear
Group of school-related NPIs (School intervention)	(Woodhouse *et al.*, 2022)^64^	Modelling Bubble quarantine with and without testing	Number of infected pupils	Bubble quarantine with testing can decrease the occurrence of outbreaks.	Some concerns in 4 out of 10 categories	Not reported	Unclear
Work- and retail-related NPIs	(Biglarbeigi *et al.*, 2021)^92^	Modelling Return of different occupational groups to employment	*R* number	If >38.5% of UK working age population return to work, the *R* number is expected to be higher than 1	Some concerns in 3 out of 10 categories	Modelled R0 1.34 (1.01,1.62) for the UK	Suggestive
Work- and retail-related NPIs	(Hill *et al.*, 2021a)^52^	Modelling Working from home, partial return to work, COVID-Secure workplace and adherence to isolation and test and trace	Peak number of cases, size and duration of outbreak, days spent in isolation	Partial return and the adherence to isolation, testing and contact tracing had an impact but the greatest was seen with work from home.	Some concerns in 5 out of 10 categories	If 40% of population worked from home median % of total population infected was 37% (19,51%).	Suggestive
Work- and retail-related NPIs	(Whitfield *et al.*, 2023)^63^	Modelling Workplace testing, distancing, office staff working from home, driver pairings	Transmission rates	Combination of physical distancing, work from home for office staff and fixed driver pairings reduce workplace outbreaks.	Some concerns in 4 out of 10 categories	Not reported	Unclear
Work- and retail-related NPIs	(Ying and O’Clery, 2021)^37^	Modelling Restriction in customer number, arrival rate, one-way system, face coverings and combinations of NPIs	Number of infectious plateaus, number of infections, chance of infection	Decreasing the maximum number of customers and customer arrival rate decrease number of infections as does use of face coverings. One-way system has less impact.	Some concerns in 4 out of 10 categories	Reducing maximum number of customers reduced number of infections by 75%	Unclear
Universal lockdown and related NPIs (UK population)	(Jarvis *et al.*, 2020)^93^	Cross-sectional Universal lockdown	Pre- and post-lockdown intervention ratio	Reduction in contacts occurred after lockdown.	Moderate	All contacts: 0.62 (0.37,0.89) Physical contacts: 0.37 (0.21, 0.52)	Suggestive
Universal lockdown and related NPIs (Scale UK)	(Albi, Pareschi and Zanella, 2021)^94^	Modelling Impact of relaxing lockdown measures (at different times; on school and work)	Infection rates	Relaxation of lockdown measures could lead to resurgence in infection rates.	Some concerns in 6 out of 10 categories	Not reported	Unclear
Universal lockdown and related NPIs (Scale UK)	(Chen and Qiu, 2021)^95^	Modelling Lockdown in combination with covering wearing, schools’ closure and quarantine	Cumulative number of cases	Covering wearing, lockdown, school closures and centralized quarantine had a significant impact.	Some concerns in 9 out of 10 categories	Not reported	Unclear
Universal lockdown and related NPIs (Scale UK)	(Chin *et al.*, 2021)^113^	Modelling Lockdown and various NPIs that limit mobility	Time varying *R* number	Lockdown had the biggest impact of all intervention in European countries	Some concerns in 5 out of 10 categories	Time varying *R* before UK lockdown 3.08 (2.32,3.78) after lockdown 0.81 (0.76,0.86)	Suggestive
Universal lockdown and related NPIs (Scale UK)	(Galanis *et al.*, 2021)^96^	Modelling Universal lockdown	*R* number	Early measures reduce infections, timing of lifting is less important	Some concerns in 8 out of 10 categories	Not reported	Unclear
Universal lockdown and related NPIs (Scale UK)	(Keeling *et al.*, 2021a)^97^	Modelling 2-week lockdowns (e.g. circuit breakers)	COVID-19 infections, hospitalizations and deaths	Impact is longer for infections than hospitals and deaths that lag behind	Some concerns in 4 out of 10 categories	Not reported	Unclear
Universal lockdown and related NPIs (Scale UK)	(Makris, 2021)^98^	Modelling Lockdown compared to social distancing	Infection induced fatality rates	Length of lockdown has a significant effect on deaths	Some concerns in 6 out of 10 categories	Not reported	Unclear
Universal lockdown and related NPIs (Scale UK)	(Mégarbane, Bourasset and Scherrmann, 2021)^99^	Modelling Lockdown compared to other strategies	Maximum rate of new cases, rate of regression	Early onset lockdown of sufficient duration most effective	Some concerns in 5 out 10 categories	Rate of regression of SARS-CoV2-susceptible individuals = 14 days in UK. Graphical outputs with confidence intervals.	Suggestive
Universal lockdown and related NPIs (Scale UK)	(Panovska-Griffiths *et al.*, 2022)^89^	Modelling Full and partial lockdowns with schools open and closed	Daily cumulative number of infection and deaths, *R* number	Once lockdown relaxed with some form of school reopening *R*, there would be increase in cases and *R* number	Some concerns in 2 out of 10 categories	Total estimated infections with relaxation of lockdown in March and April 47 500. Graphical output with confidence intervals.	Suggestive
Universal lockdown and related NPIs (Scale UK)	(Post *et al.*, 2021)^100^	Modelling Lockdown March 2020	Effective contact rate	Decrease in effective contact rate occurred when risk level was high then again after lockdown.	Some concerns in 6 out of 10 categories	First decrease in effective contact rate occurred after risk level changed to high (1.71,1.08) and second decrease 0.74 after lockdown.	Suggestive
Universal lockdown and related NPIs (Scale UK)	(van Bunnik *et al.*, 2021)^101^	Modelling Lockdown then relaxation of restrictions	Transmission rates	Important determinants of outcome were post-lockdown transmission rates, adherence to protective measures, size of vulnerable population and population immunity	Some concerns in 5 out of 10 categories	Not reported	Unclear
Universal lockdown and related NPIs (Scale UK)	(Violato, Violato and Violato, 2021)^102^	Modelling Lockdown	Mortality and infection rates	Lockdown by stringency level influenced total cases and deaths.	Some concerns in 7 out of 10 categories	Not reported	Unclear
Universal lockdown and related NPIs (Scale England)	(Arnold *et al.*, 2022)^103^	Modelling Lockdown timing and duration	COVID-19 cases, deaths, case fatality ratios	Implementing lockdown 1 or 2 weeks earlier could have reduced cases	Some concerns in 6 out of 10 categories	Cases at 1 week earlier 40 947 (30 317, 50 636) Cases at 2 weeks earlier: 10 494 (8033, 12 571)	Suggestive
Universal lockdown and related NPIs (Scale England)	(Boldea, Cornea-Madeira and Madeira, 2023)^104^	Modelling National lockdown in comparison to regional lockdowns	Timing and steepness of lockdowns	Two-week lockdown in December 2021 would have been more effective than the longer semi-lockdown	Some concerns in 8 out of 10 categories	Posterior median for steepness of NPI transition 0.63 (0.12,2.93); however, there are some concerns with clarity on uncertainty of the model	Unclear
Universal lockdown and related NPIs (Scale England)	(Didelot *et al.*, 2023)^105^	Modelling Lockdown (November 2020)	*R* number over time	Lag between mobility metrics and decline in transmission.	Some concerns in 5 out of 10 categories	Not reported	Unclear
Universal lockdown and related NPIs (Scale England)	(Dong *et al.*, 2022)^106^	Modelling Universal lockdown compared to various restrictions	Effect on predicted cases for local authority with highest predicted cases	Universal lockdown most effective, local lockdown effective in 4 local authorities only	Some concerns in 4 out of 10 categories	Not reported	Unclear
Universal lockdown and related NPIs (Scale England)	(Hinch *et al.*, 2022)^107^	Modelling Various lockdown scenarios related to duration and timing of lockdowns	COVID-19 cases and deaths avoided	Shorter lockdown would have delayed the timing of the second wave by a month but there would have been a large increase in cases and deaths in March 2021.	Some concerns in 4 out of 10 categories	Number of deaths avoided when comparing actual January–March 2022 to a lockdown of same duration but starting 1 month earlier 30 000 (24 000–38 000)	Suggestive
Universal lockdown and related NPIs (Scale England)	(Mintram *et al.*, 2022)^108^	Modelling Periodic lockdowns with and without vaccination	Hospital admissions	Periodic lockdown with vaccination was most successful	Some concerns in 2 out of 10 categories	Periodic lockdown without vaccination total mean hospital admission was 563.33 (SD 21.66). Baseline as 610.78 (SD 24.08)	Suggestive
Universal lockdown and related NPIs (Scale England)	(Muegge *et al.*, 2023)^109^	Modelling Three lockdowns March to May 2020, November 2020 and Jan to Mar 2021	COVID-19 mortality	It took 9 weeks after lockdown 1 and 2 to reduce mortality risk. Third lockdown followed tiered restrictions so does not follow same pattern	Some concerns in 3 out of 10 categories	Not reported	Unclear
Universal lockdown and related NPIs (Scale Northern Ireland)	(Abernethy and Glass, 2022)^110^	Modelling Impact of intensity and duration of lockdown measures	COVID-19 transmission, hospital admissions, ICU admission and deaths	Stronger and longer lockdowns were most effective	Some concerns in 5 out of 10 categories	Single lockdown lasting 30 days reduces deaths from 16 000 to 10 000	Unclear
Universal lockdown and related NPIs (Scale Northern Ireland)	(Kamiya *et al.*, 2023)^111^	Modelling Lockdown in Northern Ireland	Cumulative number of hospitalization	Earlier lockdown reduced hospital admission	Some concerns in 4 out of 10 categories	No numerical results but results reported in figures with confidence intervals; however, there are some concerns with clarity on uncertainty of the model	Unclear
Universal lockdown and related NPIs (Scale Scotland)	(Banks *et al.*, 2022)^112^	Modelling Impact of lockdown restrictions. Included area-based deprivation in model.	COVID-19 transmission rate and mortality	Lockdowns impact spread of disease; earlier lockdown would have reduced deaths.	Some concerns in 5 out of 10 categories	Earlier lockdown median deaths 581 (377, 1010) compared to 2722 (1294, 4050); however, there are some concerns with clarity on uncertainty of the model	Unclear
Targeted or local lockdowns	(Alsing, Usher and Crowley, 2020)^54^	Modelling Spatially targeted lockdown	Infection rate	Targeted lockdown led to strong outbreak suppression	Some concerns in 7 out of 10 categories	Not reported	Unclear
Targeted or local lockdowns	(Bittihn *et al.*, 2021)^114^	Modelling National and regional containment strategies	Days of effective national or regional lockdown and cross-region leakiness	If *R* was only slightly larger than 1 reduced restriction time of regional containment was needed.	Some concerns in 5 out of 10 categories	Not reported	Unclear
Targeted or local lockdowns	(Goscé *et al.*, 2020)^56^	Modelling Citywide lockdown with without use of testing, face coverings and contact tracing	Ratio of cumulative deaths, *R* number	Continued lockdown more effective than other strategies	Some concerns in 4 out of 10 categories	Not reported	Unclear
Targeted or local lockdowns	(Julliard, Shi and Yuan, 2023)^115^	Modelling Timing and targeting approach to lockdowns	Number of cases averted and reduction in total cases	Targeted lockdown could have contained spread of disease	Some concerns in 2 out of 10 categories	Lockdown 2 weeks earlier could have reduced cases by 20%	Unclear
Tiered restrictions	(Davies *et al.*, 2021)^84^	Modelling Tiered restrictions and circuit/fire breaker strategies in Wales and Northern Ireland	Transmission, hospital admissions, deaths	Greatest number of admission Midlands, Northeast, Yorkshire and Northwest. Tier 3 restriction caused a greater reduction in mobility. Lockdown had greater effect than circuit breaker	Some concerns in 2 out of 10 categories	Reduction in R number of 2% (0, 4) in Tier 2 and reduction by 10% (6,14) in Tier 3	Suggestive
Tiered restrictions	(Laydon *et al.*, 2021)^116^	Modelling Tiered restrictions, 2 and 3 compared to 1 or no restrictions	Real time *R* number, percentage reduction in transmission	Tier 3 produced more of a reduction in transmission than Tier 2 or Tier 1	Some concerns in 4 out of 10 categories	Tier 3 reduced transmission by 23% (21,25), Tier 2 by 6% (5,7) compared to Tier 1–0	Suggestive
Shielding	(Filipe *et al.*, 2023)^117^	Case control Shielding for those over 70 all the time and during high-risk periods	All-cause mortality (hazards ratio), HR for effect of shielding	Shielding lowers the risk of COVID-19 deaths in the shielding group than if shielding was not in place.	High	Lower risk of COVID-19 deaths in high-risk periods, HR 0.43 (0.43,0.43)	Suggestive
Shielding	(Jani *et al.*, 2021)^118^	Cohort Shielding for those at highest risk and advice for those at moderate risk	Relative risk of COVID-19 infection, hospital/ICU admission, mortality	Higher infection rate and higher risk of death in first wave of pandemic in the shielding group compared to low-risk groups.	High	COVID-19 case fatality rate RR 5.62 (4.47,7.07) compared to low-risk group	Unclear
Shielding	(Kumari *et al.*, 2021)^119^	Cross-sectional Household where someone received a letter advising to shield for 12 weeks and those shielding	Probability of reporting COVID-19 symptoms or odds ratio for positive test (in supplementary materials)	Having a household member who is shielding was associated with lower odds of positive test or symptoms, whereas no association was seen for those who received a shielding letter versus those who did not.	Moderate	OR of positive test or symptoms having a household member who is shielding 0.60 (0.38,0.94) OR of positive test or symptoms for those receiving a shielding letter 0.79 (0.50,1.23)	Unclear
Shielding	(Snooks *et al.*, 2023)^120^	Cohort Shielding for those at highest risk	COVID-19 testing rate, proportion of positive tests and proportion of known infection, COVID-19 hospital admission deaths	Inconclusive. As expected, deaths and healthcare utilization higher in (sicker) shielding vs. (healthier) non-shielding group. Odds of positive COVID-19 test result higher in non-shielding vs. shielding group, but confounded by higher rates of testing in shielding group, so difficult to interpret.	High	Odds of positive COVID-19 test in shielding versus non shielding group OR 0.716 (0.697, 0.736). OR for mortality in the shielded vs. non-shielded 3.683 (95% CI: 3.583–3.786). Proportion of recorded deaths attributable to COVID-19 15.3% (shielded) vs. 21.4% (unshielded).	Inconclusive
Shielding	(Davies *et al.*, 2020)^39^	Modelling Shielding for people 70 years and older, compared with no interventions as well as multiple NPIs	Number of cases/death, peak number of cases, deaths and ICU/non-ICU beds, time to peak cases	When used in combination with other NPIs it was most effective. Shielding of older people had the greatest impact on number of deaths.	Some concerns in 4 out of 10 categories	No numerical results on shielding but graphical output with confidence intervals	Suggestive
Shielding	(Goscé *et al.*, 2020)^56^	Modelling Shielding of people older than 60 years	Ratio of cumulative deaths	Compared to prolonged lockdown shielding alone would have results in more deaths.	Some concerns in 5 out of 10 categories	Ratio of cumulative deaths 4.5 higher	Unclear
Shielding	(Smith, Yates and Ashby, 2022)^121^	Modelling Shielding of vulnerable cases—no shielding compared to imperfect and perfect shielding	Mortality rate	Shielding reduced mortality but shielding without other interventions would have led to many avoidable deaths.	Some concerns in 4 out of 10 categories	Mortality rates no shielding 415.1 (408.5, 421.6) first wave and 87.6 (84.2, 91.1) with perfect shielding and 221.7 (217.8, 225.5) imperfect shielding.	Suggestive
Cohorting and reverse cohorting	(Bays *et al.*, 2021)^122^	Modelling Shielding with cohorting and reverse cohorting in prisons	Infection rates, case attack rate, hospitalizations	Shielding and cohorting can be used to manage outbreaks and reverse cohorting can identify incoming infections.	Some concerns in 8 out of 10 categories	Cohorting and shielding can reduce infections by 19.2% and time of peak number of infections delayed by 53%	Unclear
Cohorting and reverse cohorting	(Kaiser, Kretschmer and Leszczensky, 2021)^91^	Modelling Effects of cohorting compared to no cohorting within school environment	Proportion of outbreaks, proportion of infected students, proportion quarantined	Can reduce incidence within classroom with network-based strategies that factor in out of school contacts stopping superspreading	Some concerns in 7 out of 10 categories	Not reported	Unclear

**Table 2:** Synthesis of evidence by NPI category.

NPI category	Study characteristics (*n* studies)	Number and design of studies	Methodological quality by study design (n studies): (i) interventional, (ii) observational (iii) modelling	Relevance of studies to review question in terms of: (i) study designs, (ii) study heterogeneity	Direction of effect by study design (n studies): (i) interventional (ii) observational (iii) modelling	Assessment of precision	Certainty of evidence
Face covering use giving protection to the wearer	Interventions: type of face covering not specified (3) Settings and populations: UK adult population (1) university staff and students (1), public transport passengers (1). COVID-19 outcomes: cases (2), viral dose (1). Comparators: no NPI (2), range of scenarios (1)	3 (2 cross-sectional, 1 modelling)	(i) N/A (ii) Moderate quality cross-sectional studies (2) (iii) Some concerns reported (1)	(i) Modelling and cross-sectional studies cannot establish intervention effectiveness (ii) Some heterogeneity in study populations, COVID-19 outcomes and comparators	(i) N/A (ii) Suggestive (1), unclear (1) (iii) Unclear (1) (Authors of all 3 studies concluded that face coverings protect the wearer; however, certainty was downgraded to very low because of study heterogeneity and inadequate assessment of precision).	1/3 studies adequately assessed precision	Very low
Face covering use reducing risk of transmission	Interventions: type of face covering not specified (9), surgical masks (1) Settings and populations: school staff in Wales (1), UK population (4), NI population (2), people in public building (3). COVID-19 outcomes: cases (8), deaths (2), hospitalization (2), *R* number (1). Comparators: no NPI (5), observed population level of compliance with NPI (2), pre-pandemic level of contact intensity (1), ventilation (1), varying levels of compliance with NPI (1)	10 (1 cross-sectional, 9 modelling)	(i) N/A (ii) Moderate quality cross-sectional (1) (iii) Some concerns reported (9)	(i) Modelling and cross-sectional studies cannot establish intervention effectiveness (ii) Substantial heterogeneity in the NPI (type of face covering), study settings, COVID-19 outcomes and comparators	(i) N/A (ii) Not suggestive (1) (iii) Suggestive (3), not suggestive (1), unclear (5) (Authors of 8/9 modelling studies concluded use of face coverings was potentially protective; however, only 3 adequately assessed precision and the cross-sectional and one modelling study found no evidence of effectiveness.)	5/10 studies adequately assessed precision	Inconclusive
Physical distancing	Interventions: physical distancing (1) 2 m physical distancing (2), being in crowded room of 10 or 100 people (1) Settings and populations: UK population (2), university staff and students (1), school staff in Wales (1) COVID-19 outcomes: cases (4), hospitalization (1), deaths (1), time to peak cases (1). Comparators: no NPI (4)	4 (3 cross-sectional, 1 modelling)	(i) N/A (ii) Moderate quality cross-sectional (3) (iii) Some concerns reported (1)	(i) Modelling and cross-sectional studies cannot establish intervention effectiveness (ii) Some heterogeneity in NPI, study populations and settings, COVID-19 outcomes	(i) N/A (ii) Suggestive (1), not suggestive (1), unclear (1) (iii) Not suggestive (Of the 3 cross-sectional studies, 1 found avoiding crowded indoor spaces to be protective, 1 found no evidence that maintaining 2 m physical distancing reduced case numbers and one did not report CI. The modelling study found no evidence that physical distancing was protective.)	3/4 studies adequately assessed precision	Inconclusive
Ventilation	Interventions: natural ventilation (1), mechanical ventilation (2), increased ventilation rate (1) Settings and populations: post-secondary education settings (2), public transport (1) COVID-19 outcomes: cases (2), airborne viral dose (1) Comparators: no NPI (1), use of face coverings (1), range of scenarios (1)	3 modelling	(i) N/A (ii) N/A (iii) Some concerns reported (3)	(i) Modelling studies cannot establish intervention effectiveness (ii) Substantial heterogeneity in NPI, setting/population, COVID-19 outcomes and comparators	(i) N/A (ii) N/A (iii) Unclear (3) (Authors of all 3 studies concluded that enhanced ventilation reduces concentration of airborne virus; however, none of the studies reported effect estimates with confidence intervals)	0/3 studies adequately assessed precision	Very low
Personal and household hygiene	Interventions: hand hygiene (3), respiratory hygiene (1), cleaning surfaces (1). Settings and populations: UK population (1), university population (1), public building (1). COVID-19 outcomes: cases (3) Comparators: no NPI (3)	3 (2 cross-sectional, 1 modelling)	(i) N/A (ii) Moderate quality cross-sectional (2) (iii) Some concerns reported (1)	(i) Modelling and cross-sectional studies cannot establish intervention effectiveness (ii) Heterogeneity in NPIs and study populations/settings	(i) N/A (ii) Unclear (2) (iii) Unclear (1) (Of the 2 cross-sectional studies on hand hygiene, one concluded that it was protective, but did not provide CI, and the other found conflicting evidence depending on the definition of hand hygiene. This study also looked found that cleaning surfaces increase the risk of COVID-19 infection, a result the authors attributed to recall bias, and found no evidence that avoiding touching one’s face was protective.)	1/3 studies adequately assessed precision	Inconclusive
Contact tracing	Interventions: contact tracing, unspecified (11), smart phone-based contact tracing (1), impact of failure of contact tracing system (1) Settings and populations: population-based, UK (9), England (1), Glasgow (1), UK university (2) COVID-19 outcomes: cases (11), hospitalization (1), mortality (1), R number (4) Comparators: no NPI (4), varying levels of compliance with NPI (3), delayed NPI (1), physical distancing (1), forward vs. backward (1), range of scenarios (3)	13 (1 natural experiment, 12 modelling)	(i) High quality natural experiment (1) (ii) N/A (iii) Some concerns reported (12)	(i) 1 opportunistic natural experiment which investigated the impact on COVID-19 outcomes on a failure of the contact tracing system. Remainder were modelling studies, which cannot establish intervention effectiveness. (ii) Substantial heterogeneity in NPIs, COVID-19 outcomes and comparators.	(i) Not suggestive (1) (ii) N/A (iii) Suggestive (4), unclear (8) (Authors of all 12 modelling studies consistently reported positive, but modest impact of contact tracing on COVID-19 outcomes, provided it was well implemented and strongly adhered to. However, this was inconsistent with the findings of the only non-modelling study).	5/13 studies adequately assessed precision	Inconclusive
NHS contact tracing app	Interventions: NHS COVID-19 contact tracing app (2) Settings and populations: Population-based, England and Wales (1), Glasgow (1), COVID-19 outcomes: cases (1), R number (1) Comparators: different notification windows (1), range of scenarios (1)	2 modelling	(i) N/A (ii) N/A (iii) Some concerns reported (2)	(i) Modelling studies cannot establish intervention effectiveness (ii) Heterogeneity in COVID-19 outcomes and comparators.	(i) N/A (ii) N/A (iii) Unclear (2) (Authors of both modelling studies concluded that NHS contact tracing app has benefits in reducing adverse COVID-19 outcomes).	0/2 studies adequately assessed precision	Very low
Asymptomatic testing	Interventions: school/university/workplace-based testing (4), area-based testing (2), in combination with other NPIs (4), twice weekly (1), details not specified (4) Settings and populations: UI population-wide (2), England and Wales population-wide (1), London (1), Merthyr Tydfil and Lower Cynon Valley, Wales (1), primary schools, England (1), primary schools, unspecified (1), secondary schools, England (2), UK schools and universities (1), UK university (2), UK town (1), key workers (1), workplace (1), healthcare workers (1) COVID-19 outcomes: cases (13), hospitalization (1), ICU admission (1), mortality (2), R number (2) Comparators: no NPI (6), varying levels of compliance with NPI (1), lower testing rates (1), contact tracing and physical distancing (1), isolation of symptomatic cases (1), bubble quarantine (1), range of scenarios (4)	15 modelling	(i) N/A (ii) N/A (iii) Some concerns reported (15)	(i) Modelling studies cannot establish intervention effectiveness (ii) Substantial heterogeneity in NPIs, populations/settings, COVID-19 outcomes and comparators.	(i) N/A (ii) N/A (iii) Suggestive (8), unclear (7) (Authors of all 15 studies concluded that asymptomatic testing has benefits in reducing adverse COVID-19 outcomes)	8/15 studies adequately assessed precision	Very low
Isolation of cases	Interventions: self-isolation of symptomatic/positive cases; in combination with a variety of NPIs (lockdown, contact tracing, mass testing, use of face coverings, isolation of contacts). Settings and populations: UK population-wide (5), London (1), 4 counties in SW Wales (1), students (1) COVID-19 outcomes: cases (7), mortality (2), R number (1) Comparators: no NPI (3), varying levels of compliance with NPI (2), different levels of infectiousness (1), range of scenarios (2)	8 modelling	(i) N/A (ii) N/A (iii) Some concerns reported (8)	(i) Modelling studies cannot establish intervention effectiveness (ii) Substantial heterogeneity in NPIs, populations/settings, COVID-19 outcomes and comparators.	(i) N/A (ii) N/A (iii) Suggestive (3), unclear (5) (Authors of all 8 studies concluded that isolation of cases has benefits in reducing adverse COVID-19 outcomes).	3/8 studies adequately assessed precision	Very low
Isolation of contacts	Interventions: class quarantine; population-wide isolation of contacts. Settings and populations: UK population-wide (2), London (1), 4 counties in SW Wales (1), secondary schools (1) COVID-19 outcomes: cases (4), deaths (1) Comparators: no NPI (1), different levels of infectiousness (1), pre-pandemic level of contact intensity (1), range of scenarios (2)	5 modelling	(i) N/A (ii) N/A (iii) Some concerns reported (5)	(i) Modelling studies cannot establish intervention effectiveness (ii) Substantial heterogeneity in comparators.	(i) N/A (ii) N/A (iii) Suggestive (1), unclear (4) (Authors of all 5 studies concluded that isolation of contacts has benefits in reducing adverse COVID-19 outcomes).	1/5 studies adequately assessed precision	Very low
Test and release strategies – cases	Interventions: 5-day isolation plus daily LFD testing of confirmed cases to reduce period of isolation; 3, 5 or 7 days of isolation plus 1, 2 or 3 daily negative LFD tests required for release. Settings and populations: UK population–based (1), unspecified (1) COVID-19 outcomes: infectious cases released from isolation (2) Comparators: fixed period of isolation (2)	2 modelling	(i) N/A (ii) N/A (iii) Some concerns reported (2)	(i) Modelling studies cannot establish intervention effectiveness (ii) Minimal heterogeneity.	(i) N/A (ii) N/A (iii) Unclear (2) (Authors of both studies concluded that test and release strategies for positive cases are beneficial in reducing adverse COVID-19 outcomes).	0/2 studies adequately assessed precision	Very low
Test and release strategies – contacts	Interventions: daily contact testing with no isolation if negative (varying testing strategies in terms of number of days of isolation, days of testing, number of consecutive negative tests required). Settings and populations: UK population-based (2), England population based (2), secondary schools in England (1), primary schools in England (1) COVID-19 outcomes: cases (6) Comparators: isolation of contacts (5), isolation of symptomatic cases (1)	6 (2 RCT, 1 case–control, 3 modelling)	(i) High quality RCT (2) (ii) Moderate quality case–control (1) (iii) Some concerns reported (3)	(i) Half of the studies had highly relevant or relevant study designs (ii) Some heterogeneity in the NPI and settings/populations	(i) Suggestive (2) (ii) Suggestive (1) (iii) Not suggestive (1), unclear (2) (Both RCTs and the case–control study concluded that daily contact tracing was non-inferior to self-isolation; however, the authors of one of the modelling studies concluded that test and release strategies could not contain outbreaks, so certainty was downgraded to moderate).	4/6 studies adequately assessed precision	Moderate
NPIs that limit social contacts	Interventions: Combinations of NPIs including physical distancing and school closures (2), bubbles and forms of clustering (4) limitations in social contacts unspecified (2), roadmap out of lockdown and easing of lockdown measures (2), restriction of movement to mainland (1) Settings and populations: UK (3), England (4), Hebridean Islands in Scotland (1), Northeast London (1) COVID-19 outcomes: *R* number (1), number of cases (3) percentage increase in cumulative infections (1), probability of local outbreaks (1), transmission rate (2), COVID-19 deaths (2), COVID-19 hospitalization (1), number of excess cases and deaths (1)	9 modelling	(i) N/A (ii) N/A (iii) Some concerns reported (9)	(i) Modelling studies cannot establish intervention effectiveness (ii)Substantial heterogeneity in NPIs, populations/settings, COVID-19 outcomes and comparators.	(i) N/A (ii) N/A (iii) Suggestive (2), not suggestive (1), unclear (6) (Authors concluded that reducing contacts would reduce COVID-19 outcomes)	4/9 studies adequately assessed precision	Very low
School-related NPIs	Interventions: School closures (2), return to school interventions with partial and full reopening and various class sizes (7), within school interventions (6) such as teaching indoors/outdoors, bubbles, cohorting Settings and populations: UK (7), Wales (1), England (5), England/Wales/Northern Ireland (1), stated type of school: secondary schools (1), primary schools (3), primary and secondary (3), children aged 14–15 years (1) COVID-19 outcomes: COVID-19 cases (4), COVID-19 deaths (2), ICU beds and non-ICU beds in peak weeks (1), transmission rates (3), hospital admissions (1), number of schools with one or more infected person (1), *R* number (4), secondary infections (1), transmission between schools (1), daily cumulative number of infections (2) and deaths (2), percentage of infection and deaths (1), proportion of outbreaks (1), proportion of infected students (1), proportion quarantined (1), testing rate (1), number of absences in school (2), number of infected pupils (1)	15 (1 cross-sectional, 14 modelling)	(i) N/A (ii) Moderate quality cross-sectional (iii) Some concerns reported (14)	(i)Modelling studies cannot establish intervention effectiveness (ii)Substantial heterogeneity in NPIs, populations/settings, COVID-19 outcomes and comparators.	(i) N/A (ii) Not suggestive (1) (iii) Suggestive (9), unclear (5) (Authors concluded schools closures reduced the risk of COVID-19 and reopening of schools increased the risk. Partial reopening and using school bubbles often with testing could reduce the risk.)	11/15 studies adequately assessed precision	Low
Work- and retail-related NPIs	Interventions: Return to work of different occupational groups (1), working from home (1), COVID-19 secure workplace measures (2), workplace testing (2), paired delivery drivers (1), restricted customer numbers (1), one-way system (1), use of face coverings (1) Settings and populations: UK (2), home delivery sector (1), retail store (1) COVID-19 outcomes: *R* number (1), peak number of cases (1), size and duration of outbreak (1), days spent in isolation (1), transmission rate (1), number of infectious plateaus (1), chance of infection (1).	4 modelling	(i) N/A (ii) N/A (iii) Some concerns reported (4)	(i) Modelling studies cannot establish intervention effectiveness (ii) Substantial heterogeneity in NPIs, populations/settings, COVID-19 outcomes and comparators.	(i) N/A (ii) N/A (iii) Suggestive (2), unclear (2) (Authors concluded that measures that reduced the number returning to work would reduce COVID-19 outcomes)	2/4 studies adequately assessed precision	Very low
Universal lockdown	Interventions: Lockdown (15), relaxing lockdown measures (2), lockdown in combination with other NPIs (2), 2-week circuit breaker lockdown (2), partial lockdown (1) Settings and populations: UK (12), England (7), Northern Ireland (2), Scotland (1) COVID-19 outcomes: Pre- and post-intervention ratio (1) infection rates (2), cumulative number of cases (2), time varying *R* number (2), *R* number (2), COVID-19 infections (2), cumulative number of cases (2), hospitalizations (3), deaths (8), fatality rates (1), maximum rate of new cases (1), rate of regression (1), effective contact rate (1), transmission rate (3), timing and steepness of lockdown (1), effect on predicted cases (1), cases and deaths avoided (1), cumulative number of hospitalizations (1)	22 (1 cross-sectional, 21 modelling)	(i)N/A (ii) Moderate quality cross-sectional (iii) Some concerns reported (21)	(i) Modelling studies cannot establish intervention effectiveness (ii) Substantial heterogeneity in NPIs, populations/settings, COVID-19 outcomes and comparators.	(i) N/A (ii) Suggestive (1) (iii) Suggestive (8), unclear (14) (Authors concluded all NPIs related to lockdown would reduce COVID-19 outcomes).	11/22 studies adequately assessed precision	Low
Targeted or local lockdown	Interventions: Spatially targeted lockdown, national and regional containment (2), citywide lockdown with other NPIs (1), timing and targeting local lockdowns (1) Settings and populations: England (2), London (2) COVID-19 outcomes: Infection rate (1), days of effective national and regional lockdown (1), cross-region leakiness (1), ratio of cumulative deaths (1), *R* number (1)	4 modelling	(i) N/A (ii) N/A (iii) Some concerns reported (4)	(i) Modelling studies cannot establish intervention effectiveness (ii) Moderate heterogeneity in NPIs, populations/settings and COVID-19 outcomes.	(i) N/A (ii) N/A (iii) Unclear (4) (Authors had varied conclusions based on their models, each had different COVID-19 outcomes)	0/4 studies adequately assessed precision	Very low
Tiered restrictions	Interventions: Tiered restrictions and circuit breaker strategies (1), Tier 2 and 3 restrictions compared to tier 1 (1) Settings and populations: UK (1), England (1), Wales (1) COVID-19 outcomes: Transmission rate (1), hospital admissions deaths (1), real time *R* number (1), percentage reduction in transmission (1)	2 modelling	(i) N/A (ii) N/A (iii) Some concerns reported (2)	(i) Modelling studies cannot establish intervention effectiveness (ii) Moderate heterogeneity in NPIs, populations/settings and COVID-19 outcomes	(i) N/A (ii) N/A (iii) Suggestive (2) (Authors concluded there was a reduction in COVID-19 outcomes with higher tiers).	2/2 studies adequately assessed precision	Very low
Shielding	Interventions: Shielding for those at highest risk (2), shielding for those over 70 years (1), shielding for those over 60 years (1), shielding of vulnerable cases (1), households who received a letter to shield (1) Settings and populations: UK (2), England (1), Wales (1), West of Scotland (1), London (1), Liverpool (1), COVID-19 outcomes: Morality rate (1), ratio of cumulative deaths (1), number of cases and deaths (1), peak number of cases and deaths (1), number of ICU and non-ICU beds (1), probability of reporting COVID-19 symptoms (1), odds ratio of positive test (1), COVID-19 mortality (1), COVID-19 infection rate (1), hospital and ICU admission (1), COVID-19 testing rate (1), proportion of positive tests and proportion of known infections (1).	7 (2 cohort, 1 case–control, 1 cross-sectional, 3 modelling)	(i) N/A (ii) High-quality cohort (2), high-quality case control (1), moderated quality cross-sectional (1) (iii) Some concerns reported (3)	(i) 2 cohort studies and 1 case control study using hospital records. Remaining 4 studies were modelling and cross-sectional in design, which cannot establish intervention effectiveness. (ii) Moderate heterogeneity in NPIs, populations/settings and COVID-19 outcomes	(i) N/A (ii) Suggestive (1), unclear (3) (iii) Suggestive (2), unclear (1) (5/7 studies reported shielding would reduce COVID-19 outcomes. One cohort study found higher mortality in shielded group; however, this study period was only 3 months with shielding lists available at the midpoint of the study compared to 12 and 14 months for the other observational studies)	6/7 studies adequately assessed precision	Inconclusive
Cohorting	Interventions: Cohorting (2), Reverse cohorting (1) Settings and populations: School (1), prison (1) COVID-19 outcomes: Infection rates (1), case attack rate (1), hospitalizations (1), proportion of outbreaks (1), proportion of infected students (1), proportion quarantined (1)	2 modelling	(i) N/A (ii) N/A (iii) Some concerns reported (2)	(i) Modelling studies cannot establish intervention effectiveness (ii) Substantial heterogeneity in NPIs, populations/settings, COVID-19 outcomes and comparators.	(i) N/A (ii) N/A (iii) Unclear (2) (Authors concluded cohorting could be effective at reducing COVID-19 outcomes)	0/2 studies adequately assessed precision	Very low

The only NPI category evaluated as being effective with a moderate level of evidence certainty was test and release strategies for the contacts of positive cases. Although this NPI category included two high-quality RCTs, we downgraded evidence certainty from high to moderate because of heterogeneity in how these strategies were implemented in study settings, inadequate assessment of precision in two studies and the conclusion by one modelling study that this strategy would not contain outbreaks.

A low level of evidence certainty was assigned to school-related NPIs and universal lockdown. Both included one cross-sectional study with the remainder being modelling studies. Although all studies had the same direction of effect (suggesting that these NPIs were protective), we found heterogeneity in study characteristics and had concerns about methodological quality and inadequate assessment of precision.

A very low level of evidence certainty was assigned for face covering used to protect the wearer, ventilation, the NHS contact tracing app, asymptomatic testing, isolation of cases and contacts, test and release strategies for cases, NPIs that limit social contacts, work- and retail-related NPIs, tiered restrictions and cohorting. In all but one case, this was due to the NPI category including only modelling studies. For face coverings to protect the wearer, evidence certainty was downgraded to very low because of heterogeneity in study characteristics, failure to specify the type of face covering and inadequate assessment of precision.

We found inconclusive evidence for six NPI categories: the use of face coverings to reduce transmission, physical distancing, personal and household hygiene, contact tracing, targeted or local lockdown and shielding to reduce the risk of COVID-19 transmission. The reason for the inconclusive classification was a discrepancy in the direction of effect for at least one of the observational studies within each category. The studies also had other significant limitations. For example, only 1 of 10 studies specified the type of face covering under investigation.^33^

## Discussion

### Main findings of this study

We found 97 studies on 20 different categories of NPIs, as implemented in the UK during the COVID-19 pandemic, including 3 interventional, 9 observational and 85 modelling studies. The included studies were highly heterogeneous and were conducted under pandemic conditions when it was imperative to make predictions at speed about the potential impact of policy decisions. Most of the modelling studies did not report numerical effect estimates with CI but instead often reported graphs with different scenarios with and without the use of one or various NPIs. Thus, our findings report the direction, but not magnitude, of the likely effects of NPIs on COVID-19 outcomes.

For six NPI categories (the use of face coverings to reduce the risk of COVID-19 transmission, physical distancing, personal/household hygiene, contact tracing, targeted/local lockdown and shielding) the level of certainty in the evidence was considered inconclusive due to inconsistency in the direction of effect found by the authors. However, we were able to identify a moderate level of certainty for test and release strategies for case contacts (largely due to two high-quality RCTs). Evidence for the effectiveness of the remaining 13 NPI categories was assessed as low or very low certainty because of study design limitations, heterogeneity in study characteristics and NPI implementation and lack of precision estimation.

### What is already known on this topic

Our findings are consistent with those of global systematic reviews of observational and interventional studies.^123^^,^^124^ Talic *et al*. found evidence for the effectiveness of face coverings, physical distancing and testing followed by isolation,^123^ with moderate to high/critical risk of bias and high levels of study heterogeneity, precluding meta-analysis. A recent review of global systematic reviews found only 8 of 94 reviews to have moderate to high confidence ratings.^125^ It found low certainty of evidence for the effectiveness of multicomponent measures and active surveillance and very low certainty for travel, personal protective and environmental measures. Comparison with international studies is difficult, as multiple NPIs tended to be implemented together, making it challenging to isolate the effect of a single measure. Secondly measures such as closing of borders for long periods, as implemented in Australia,^126^ or provision of free masks and hand sanitizers for the entire population, as implemented in Singapore,^127^ may not be feasible in the UK.

### What this study adds

To the best of our knowledge, our study is the only one to synthesize all the available evidence, including from modelling studies, on NPIs as implemented in the UK. We conducted a comprehensive literature search up until January 2024, used a systematic approach to study selection, quality appraisal and data analysis and synthesized evidence from modelling as well as interventional and observational studies. A key advantage of this approach is that it allowed for the inclusion of 11 additional NPI categories for which no interventional or observational studies have been published in UK populations (e.g. ventilation, asymptomatic testing).

Consistent with other reviews,^124^^,^^125^ we found the validity and reliability of the available evidence to support the effectiveness of individual NPIs to control the spread of COVID-19 to be weak and not to provide robust evidence to inform future pandemic preparedness. The main lesson from this review is the need to improve evidence generation to support future pandemic decision-making, including building rapid evaluation into the response to pandemic and other public health emergencies. This includes the development of ‘sleeper’ study platforms and protocols,^128^ which can be activated during an epidemic or pandemic,^129^ such as the COVID-19 rapid survey of adherence to interventions and responses (CORSAIR study).^130^ Another approach is the delivery of rapid adaptive trials for the simultaneous testing of various NPIs, such as the rapid adaptive trials for pharmaceutical interventions PRINCIPLE^131^ and PANORAMIC.^132^ To facilitate rapid research and evaluation during public health emergencies, pandemic preparedness plans should embed processes for incorporating rapid data governance and ethical approvals,^133^ including the design of ethical trials (for instance, of multiple NPIs) and appropriate development of sleeper protocols that would undergo ethical approval in advance. It is also imperative to invest in data systems and develop routine health data sources like Open Safely,^134^ which would allow for a clearer indication of the effectiveness of interventions in near real-time.

### Limitations of this study

As this was a rapid review, some processes were truncated, which could have introduced bias (e.g. data extraction was not conducted in duplicate and we adapted a critical appraisal tool for modelling studies). Focusing exclusively on the UK reduced some of the heterogeneity due to differences between countries but resulted in the exclusion of potentially high-quality and relevant evidence from other populations. By excluding laboratory studies on the physical properties of the SARS-CoV-2 virus and physical studies on the behaviour of airborne particles, we may have missed important studies that could have provided evidence on mechanistic aspects and efficacy of NPIs. Most of the included studies had a high risk of bias. Only three were RCTs but even these are at risk of residual confounding from environmental and behavioural factors. Finally, our review has considered the impact of NPIs on reducing COVID-19 outcomes, but this needs to be balanced against the potential adverse economic, political or social effects associated with the adoption of NPIs during the COVID-19 pandemic.^135^

## Conclusion

Our review found that evidence for the effectiveness of individual NPIs as implemented in the UK to control the spread of COVID-19 is weak. The best available evidence was for test and release strategies for case contacts (moderate certainty), which was suggestive of a protective effect. Although evidence for school-related NPIs and universal lockdown was also suggestive of a protective effect, this evidence was considered low certainty. Evidence certainty for the remaining NPIs was very low or inconclusive. There were limitations in study designs and methodological quality, heterogeneity in study characteristics and challenges in isolating the effects of single interventions, in the context of multiple interventions being implemented simultaneously. These results do not necessarily reflect a lack of effectiveness of packages of NPIs implemented in the UK. However, they highlight the need to build evaluation into the design of public health interventions to improve evidence generation in order to support future pandemic decision-making.

## Supplementary Material

Supplementary_materials_Effectivenesss_of_COVID-19_NPIs_in_the_UK_fdaf017

## Data Availability

Data extraction and quality appraisal for all included studies can be obtained by contacting the corresponding author.

## Disclaimer

the views expressed in this article are those of the authors and are not necessarily those of United Kingdom Health Security Agency or the Department of Health and Social Care. EMcS’ role in this research is independent of her PhD research project which is funded by the Legal & General Group (research grant to establish the independent Advanced Care Research Centre at University of Edinburgh). The funders had no role in conduct of the study, interpretation or the decision to submit for publication. The views expressed are those of the authors and not necessarily those of Legal & General.

## Notes

**T. Ashcroft,** UNCOVER Project Manager

**E. McSwiggan,** UNCOVER Project Manager

**E. Agyei-Manu,** UNCOVER Reviewer

**M. Nundy,** UNCOVER Reviewer

**N. Atkins**, UNCOVER Reviewer

**J.R. Kirkwood,** UNCOVER Reviewer

**M. Ben Salem Machiri,** UNCOVER Reviewer

**V. Vardhan,** UNCOVER Reviewer

**B. Lee,** UNCOVER Reviewer

**E. Kubat,** UNCOVER Reviewer

**S. Ravishankar,** UNCOVER Reviewer

**P. Krishan,** UNCOVER Reviewer

**U. De Silva,** UNCOVER Reviewer

**E.O. Iyahen,** UNCOVER Reviewer

**J. Rostron,** UNCOVER Reviewer

**A. Zawiejska,** UNCOVER Reviewer

**K. Ogarrio,** UNCOVER Reviewer

**M. Harikar,** UNCOVER Reviewer

**S. Chishty,** UNCOVER Reviewer

**D. Mureyi**, UNCOVER Reviewer

**B. Evans,** Senior Evidence Reviewer

**D. Duval,** Evidence Review Lead

**S. Carville,** Head of Evidence

**S. Brini,** Principal Evidence Reviewer

**J. Hill**, Information Scientist

**M. Qureshi**, Evidence Reviewer

**Z. Simmons**, Senior Evidence Reviewer

**I. Lyell,** Public Health Specialty Registrar

**T. Kavoi,** Public Health Specialty Registrar

**M. Dozier,** Lead Academic Support Librarian

**G. Curry**, Reader in Race, Ethnicity and Health

**J.M. Ordóñez-Mena,** Senior Medical Statistician

**S. de Lusignan,** Professor of Primary Care and Clinical Informatics

**A. Sheikh,** Professor of Primary Care Research and Development

**E. Theodoratou,** Professor of Cancer Epidemiology and Global Health

**R. McQuillan,** Reader
