# Supplementary material for: Effectiveness of non-pharmaceutical interventions as implemented in the UK during the COVID-19 pandemic: a rapid review
Source: J Public Health (Oxf). 2025 Mar 4;47(2):268–302. doi: 10.1093/pubmed/fdaf017 (PMC12123321; doi:10.1093/pubmed/fdaf017)
Supplement: Supplementary_materials_Effectivenesss_of_COVID-19_NPIs_in_the_UK_fdaf017 [file supplementary_materials_effectivenesss_of_covid-19_npis_in_the_uk_fdaf017.docx]

# Effectiveness of COViD-19 NPIs as implemented in the UK: Supplementary materials

Contents

Appendix 1: Methods

1A: Search strategies…………………………………………………………………………………………………………..2

1B: Quality appraisal methodology for interventional and observational studies………….….14

1C: Quality appraisal methodology for modelling studies…………………………………………….……15

1D: Decision algorithm for level of certainty of evidence……………………………………………….….18

Appendix 2: Results

2A: Table of characteristics of included studies………………………………………………………………….19

2B: Table with quality appraisal of observational and interventional studies…………………….

2C: Table with quality appraisal of modelling studies………………………………………………………..

**1A Search strategies**

Databases searched

The following databases were searched:

- Ovid MEDLINE(R) ALL (1946 to 29 December 2023)
- Embase (1974 to 29 December 2023)
- NIH Covid Portfolio (searches date limited from 28/02/2023 to 02/01/2024)
- Corona Central (date limited from 24/02/23 to 02/01/24)

**Database:** **Ovid MEDLINE(R) ALL <1946 to December 29, 2023>**

**Date of search:** 02/01/2024

**Search Strategy:**
**1**  exp SARS-CoV-2/ (163534)
**2**  exp COVID-19/ (250530)
**3**  (corona* adj1 (virus* or viral*)).tw,kw,kf. (6587)
**4**  (CoV not (Coefficien* or "co-efficien*" or covalent* or Covington* or covariant* or covarianc* or "cut-off value*" or "cutoff value*" or "cut-off volume*" or "cutoff volume*" or "combined optimi?ation value*" or "central vessel trunk*" or CoVR or CoVS)).tw,kw,kf. (134000)
**5**  (coronavirus* or 2019nCoV* or 19nCoV* or "2019 novel*" or Ncov* or "n-cov" or "SARS-CoV-2*" or "SARSCoV-2*" or SARSCoV2* or "SARS-CoV2*" or "severe acute respiratory syndrome*" or COVID*2).tw,kw,kf. (410820)
**6**  exp COVID-19 Vaccines/ (24612)
**7**  exp COVID-19 Testing/ (12034)
**8**  or/1-7 (418732)
**9**  Contact Tracing/ (6338)
**10**  ((contact or source or infection or patient or case) adj2 (screen* or notificat* or trac* or investig*)).tw,kf. (88798)
**11**  (NPI* or ((non-pharm* or nonpharm*) adj intervention*) or "public health measure*" or (prevent* adj2 measure*)).tw,kf. (69812)
**12**  9 or 10 or 11 (161280)
**13**  Quarantine/ (6328)
**14**  Social Isolation/ (16428)
**15**  (quarantin* or isolat*).ti,kf. or (quarantin* or isolat*).ab. /freq=2 (681233)
**16**  13 or 14 or 15 (695605)
**17**  exp *COVID-19 Testing/ (2987)
**18**  Point-of-Care Testing/ or Self-Testing/ (4666)
**19**  Reagent Kits, Diagnostic/ (17608)
**20**  ("lateral flow" or LFT or LFA or LFD or LFIA).tw,kf. (15002)
**21**  ((COVID or Corona*) adj2 test*).tw,kf. (7841)
**22**  17 or 18 or 19 or 20 or 21 (46417)
**23**  (lockdown* or lock-down*).ti,kf. or (lockdown* or lock-down*).ab. /freq=2 (13121)
**24**  ((stay adj2 home) or shielding).tw,kf. (20508)
**25**  Physical Distancing/ (2389)
**26**  ((physical* or social*) adj distan*).tw,kf. (15404)
**27**  ((social* or societal* or gathering* or meeting* or event*) adj3 (restrict* or prohibit* or limit* or ban* or cancel*)).tw,kf. (14909)
**28**  (tier* adj2 restric*).tw,kf. (27)
**29**  23 or 24 or 25 or 26 or 27 or 28 (61076)
**30**  exp Disinfectants/ or Disinfection/ (89387)
**31**  ((environment* or surface* or home* or house* or workplace*) adj3 disinfect*).tw,kf. (3192)
**32**  ((environment* or surface* or home* or house* or workplace*) adj3 clean*).tw,kf. (8534)
**33**  ((environment* or surface* or home* or house* or workplace*) adj3 decontaminat*).tw,kf. (1383)
**34**  deep clean*.tw,kf. (73)
**35**  30 or 31 or 32 or 33 or 34 (99922)
**36**  exp Hand Hygiene/ (8250)
**37**  (hand wash* or handwash*).tw,kf. (6764)
**38**  hand saniti*.tw,kf. (1054)
**39**  ((hand or personal) adj hygiene).tw,kf. (9303)
**40**  36 or 37 or 38 or 39 (18277)
**41**  (facemask* or mask*).tw,kf. (105945)
**42**  Masks/ (7504)
**43**  (face adj2 (cover* or protect*)).tw,kf. (1166)
**44**  (mouth adj2 (cover* or protect*)).tw,kf. (385)
**45**  (nose adj2 (cover* or protect*)).tw,kf. (159)
**46**  (respirator or respirators).tw,kf. (7067)
**47**  41 or 42 or 43 or 44 or 45 or 46 (113948)
**48**  ((universit* or college* or school*) adj3 clos*).tw,kf. (3479)
**49**  ((office* or work*) adj3 clos*).tw,kf. (5987)
**50**  (home* adj3 work*).tw,kf. (11203)
**51**  ((hospitalit* or restaurant* or cafe* or venue* or shop* or retail* or hotel* or leisure or gym* or cinema* or theatre* or theater*) adj3 clos*).tw,kf. (521)
**52**  48 or 49 or 50 or 51 (20806)
**53**  border health.tw,kf. (276)
**54**  (Travel* adj5 (ban* or restrict*)).tw,kf. (2040)
**55**  (Border* adj5 (control* or restrict*)).tw,kf. (2240)
**56**  ((entrance or Entry) adj5 restrict*).tw,kf. (1079)
**57**  (Movement* adj5 restrict*).tw,kf. (6033)
**58**  53 or 54 or 55 or 56 or 57 (11419)
**59**  ((ventilation or ventilated) and (transmission* or distanc* or dispers* or aerosol* or airborne or air qualit* or indoor air)).tw,kf. (9447)
**60**  Ventilation/ (6380)
**61**  (air flow* or airflow* or aerodynamic* or air condition*).tw,kf. (38973)
**62**  Air Conditioning/ (2895)
**63**  (air filter* or air purif* or air filtration).tw,kf. (2973)
**64**  Air Filters/ (620)
**65**  (air chang* or air exchang*).tw,kf. (1953)
**66**  (air adj3 (recondition* or re condition*)).tw,kf. (1)
**67**  (air adj3 replac*).tw,kf. (416)
**68**  (indoor air adj3 qualit*).tw,kf. (3132)
**69**  HVAC.tw,kf. (625)
**70**  Air Microbiology/ (8323)
**71**  59 or 60 or 61 or 62 or 63 or 64 or 65 or 66 or 67 or 68 or 69 or 70 (65230)
**72**  12 or 16 or 22 or 29 or 35 or 40 or 47 or 52 or 58 or 71 (1245446)
**73**  8 and 72 (72403)
**74**  exp United Kingdom/ (392514)
**75**  (national health service* or nhs*).ti,ab,in. (281669)
**76**  (english not ((published or publication* or translat* or written or language* or speak* or literature or citation*) adj5 english)).ti,ab. (51735)
**77**  (gb or "g.b." or britain* or (british* not "british columbia") or uk or "u.k." or united kingdom* or (england* not "new england") or northern ireland* or northern irish* or scotland* or scottish* or ((wales or "south wales") not "new south wales") or welsh*).ti,ab,jw,in. (2505312)
**78**  (bath or "bath's" or ((birmingham not alabama*) or ("birmingham's" not alabama*) or bradford or "bradford's" or brighton or "brighton's" or bristol or "bristol's" or carlisle* or "carlisle's" or (cambridge not (massachusetts* or boston* or harvard*)) or ("cambridge's" not (massachusetts* or boston* or harvard*)) or (canterbury not zealand*) or ("canterbury's" not zealand*) or chelmsford or "chelmsford's" or chester or "chester's" or chichester or "chichester's" or coventry or "coventry's" or derby or "derby's" or (durham not (carolina* or nc)) or ("durham's" not (carolina* or nc)) or ely or "ely's" or exeter or "exeter's" or gloucester or "gloucester's" or hereford or "hereford's" or hull or "hull's" or lancaster or "lancaster's" or leeds* or leicester or "leicester's" or (lincoln not nebraska*) or ("lincoln's" not nebraska*) or (liverpool not (new south wales* or nsw)) or ("liverpool's" not (new south wales* or nsw)) or ((london not (ontario* or ont or toronto*)) or ("london's" not (ontario* or ont or toronto*)) or manchester or "manchester's" or (newcastle not (new south wales* or nsw)) or ("newcastle's" not (new south wales* or nsw)) or norwich or "norwich's" or nottingham or "nottingham's" or oxford or "oxford's" or peterborough or "peterborough's" or plymouth or "plymouth's" or portsmouth or "portsmouth's" or preston or "preston's" or ripon or "ripon's" or salford or "salford's" or salisbury or "salisbury's" or sheffield or "sheffield's" or southampton or "southampton's" or st albans or stoke or "stoke's" or sunderland or "sunderland's" or truro or "truro's" or wakefield or "wakefield's" or wells or westminster or "westminster's" or winchester or "winchester's" or wolverhampton or "wolverhampton's" or (worcester not (massachusetts* or boston* or harvard*)) or ("worcester's" not (massachusetts* or boston* or harvard*)) or (york not ("new york*" or ny or ontario* or ont or toronto*)) or ("york's" not ("new york*" or ny or ontario* or ont or toronto*))))).ti,ab,in. (1796880)
**79**  (bangor or "bangor's" or cardiff or "cardiff's" or newport or "newport's" or st asaph or "st asaph's" or st davids or swansea or "swansea's").ti,ab,in. (72545)
**80**  (aberdeen or "aberdeen's" or dundee or "dundee's" or edinburgh or "edinburgh's" or glasgow or "glasgow's" or inverness or (perth not australia*) or ("perth's" not australia*) or stirling or "stirling's").ti,ab,in. (264359)
**81**  (armagh or "armagh's" or belfast or "belfast's" or lisburn or "lisburn's" or londonderry or "londonderry's" or derry or "derry's" or newry or "newry's").ti,ab,in. (34972)
**82**  or/74-81 (3143783)
**83**  (exp africa/ or exp americas/ or exp antarctic regions/ or exp arctic regions/ or exp asia/ or exp australia/ or exp oceania/) not (exp United Kingdom/ or europe/) (3375175)
**84**  82 not 83 (2974881)
**85**  73 and 84 (9750)
**86**  limit 85 to dt=20230228-20240102 (1257)
**87**  exp animals/ not humans.sh. (5182085)
**88**  86 not 87 (1254)

**Database: Embase <1974 to 2023 December 29>**

**Date of search:** 02/01/2024

**Search Strategy:**
**1**  exp severe acute respiratory syndrome coronavirus 2/ (106768)
**2**  coronavirus disease 2019/ (369843)
**3**  interventional coronavirus disease 2019/ (22)
**4**  (corona* adj1 (virus* or viral*)).tw,kw. (6637)
**5**  (CoV not (Coefficien* or co-efficien* or covalent* or covington or covariant* or covarianc* or "cut-off value*" or "cutoff value*" or "cut-off volume*" or "cutoff volume*" or "combined optimi?ation value*" or "central vessel trunk" or CoVR or CoVS)).tw,kw. (136078)
**6**  (coronavirus* or 2019nCoV* or 19nCoV* or "2019 novel*" or Ncov* or "n-cov" or "SARS CoV-2*" or "SARSCoV-2*" or SARSCoV2* or "SARS-CoV2*" or "severe acute respiratory syndrome*" or COVID*2).tw,kw. (461327)
**7**  COVID-19 Testing/ (8296)
**8**  exp SARS-CoV-2 vaccine/ (43462)
**9**  or/1-8 (498017)
**10**  exp contact examination/ (8782)
**11**  ((contact or source or infection or patient or case) adj2 (screen* or notificat* or trac* or investig*)).tw,kf. (131725)
**12**  (NPI* or ((non-pharm* or nonpharm*) adj intervention*)).tw,kf. (18986)
**13**  10 or 11 or 12 (155473)
**14**  exp *quarantine/ (2170)
**15**  *social isolation/ (7948)
**16**  (quarantin* or isolat*).ti,kf. or (quarantin* or isolat*).ab. /freq=2 (781816)
**17**  14 or 15 or 16 (786059)
**18**  *COVID-19 Testing/ (2634)
**19**  exp *"point of care testing"/ (8107)
**20**  self-testing/ (1205)
**21**  exp *infectious disease test kit/ (6604)
**22**  ("lateral flow" or LFT or LFA or LFD or LFIA).tw,kf. (20476)
**23**  lateral flow immunochromatography/ (2152)
**24**  ((COVID or Corona*) adj2 test*).tw,kf. (10562)
**25**  18 or 19 or 20 or 21 or 22 or 23 or 24 (47462)
**26**  (lockdown* or lock-down*).ti,kf. or (lockdown* or lock-down*).ab. /freq=2 (13053)
**27**  ((stay adj2 home) or shielding).tw,kf. (23459)
**28**  *social distancing/ (1125)
**29**  mass gathering/ (359)
**30**  ((physical* or social*) adj distan*).tw,kf. (15885)
**31**  ((social* or societal* or gathering* or meeting* or event*) adj3 (restrict* or prohibit* or limit* or ban* or cancel*)).tw,kf. (20416)
**32**  (tier* adj2 restric*).tw,kf. (31)
**33**  26 or 27 or 28 or 29 or 30 or 31 or 32 (69568)
**34**  exp *disinfectant agent/ (206126)
**35**  *disinfection/ (13187)
**36**  *cleaning/ (3860)
**37**  ((environment* or surface* or home* or house* or workplace*) adj3 disinfect*).tw,kf. (3691)
**38**  ((environment* or surface* or home* or house* or workplace*) adj3 clean*).tw,kf. (10035)
**39**  ((environment* or surface* or home* or house* or workplace*) adj3 decontaminat*).tw,kf. (1514)
**40**  deep clean*.tw,kf. (145)
**41**  34 or 35 or 36 or 37 or 38 or 39 or 40 (231675)
**42**  exp *hand washing/ (5453)
**43**  (hand wash* or handwash*).tw,kf. (8659)
**44**  hand saniti*.tw,kf. (1380)
**45**  ((hand or personal) adj hygiene).tw,kf. (13582)
**46**  exp *personal hygiene/ (10657)
**47**  42 or 43 or 44 or 45 or 46 (32442)
**48**  (facemask* or mask*).tw,kf. (131583)
**49**  exp *face mask/ or exp filtering facepiece respirator/ (5700)
**50**  (face adj2 (cover* or protect*)).tw,kf. (1242)
**51**  (mouth adj2 (cover* or protect*)).tw,kf. (391)
**52**  (nose adj2 (cover* or protect*)).tw,kf. (213)
**53**  (respirator or respirators).tw,kf. (7010)
**54**  48 or 49 or 50 or 51 or 52 or 53 (139656)
**55**  ((universit* or college* or school*) adj3 clos*).tw,kf. (3555)
**56**  ((office* or work*) adj3 clos*).tw,kf. (8735)
**57**  (home* adj3 work*).tw,kf. (13735)
**58**  ((hospitalit* or restaurant* or cafe* or venue* or shop* or retail* or hotel* or leisure or gym* or cinema* or theatre* or theater*) adj3 clos*).tw,kf. (600)
**59**  school closure/ (697)
**60**  55 or 56 or 57 or 58 or 59 (26391)
**61**  border health.tw,kf. (355)
**62**  travel restriction/ (1278)
**63**  (Travel* adj5 (ban* or restrict*)).tw,kf. (2144)
**64**  (Border* adj5 (control* or restrict*)).tw,kf. (2726)
**65**  ((entrance or Entry) adj5 restrict*).tw,kf. (1322)
**66**  (Movement* adj5 restrict*).tw,kf. (7448)
**67**  61 or 62 or 63 or 64 or 65 or 66 (14526)
**68**  ((ventilation or ventilated) and (transmission* or distanc* or dispers* or aerosol* or airborne or air qualit* or indoor air)).tw,kf. (13453)
**69**  *air conditioning/ (5947)
**70**  (air flow* or airflow* or aerodynamic* or air condition*).tw,kf. (53039)
**71**  (air filter* or air purif* or air filtration).tw,kf. (3525)
**72**  exp air filter/ (2570)
**73**  (air chang* or air exchang*).tw,kf. (2707)
**74**  (air adj3 (recondition* or re condition*)).tw,kf. (4)
**75**  (air adj3 replac*).tw,kf. (490)
**76**  (indoor air adj3 qualit*).tw,kf. (4381)
**77**  HVAC.tw,kf. (790)
**78**  airborne virus/ (394)
**79**  exp airborne transmission/ (1538)
**80**  68 or 69 or 70 or 71 or 72 or 73 or 74 or 75 or 76 or 77 or 78 or 79 (79488)
**81**  13 or 17 or 25 or 33 or 41 or 47 or 54 or 60 or 67 or 80 (1532052)
**82**  9 and 81 (75864)
**83**  exp United Kingdom/ (469258)
**84**  (national health service* or nhs*).ti,ab,in,ad. (482596)
**85**  (english not ((published or publication* or translat* or written or language* or speak* or literature or citation*) adj5 english)).ti,ab. (62159)
**86**  (gb or "g.b." or britain* or (british* not "british columbia") or uk or "u.k." or united kingdom* or (england* not "new england") or northern ireland* or northern irish* or scotland* or scottish* or ((wales or "south wales") not "new south wales") or welsh*).ti,ab,jx,in,ad. (3776208)
**87**  (bath or "bath's" or ((birmingham not alabama*) or ("birmingham's" not alabama*) or bradford or "bradford's" or brighton or "brighton's" or bristol or "bristol's" or carlisle* or "carlisle's" or (cambridge not (massachusetts* or boston* or harvard*)) or ("cambridge's" not (massachusetts* or boston* or harvard*)) or (canterbury not zealand*) or ("canterbury's" not zealand*) or chelmsford or "chelmsford's" or chester or "chester's" or chichester or "chichester's" or coventry or "coventry's" or derby or "derby's" or (durham not (carolina* or nc)) or ("durham's" not (carolina* or nc)) or ely or "ely's" or exeter or "exeter's" or gloucester or "gloucester's" or hereford or "hereford's" or hull or "hull's" or lancaster or "lancaster's" or leeds* or leicester or "leicester's" or (lincoln not nebraska*) or ("lincoln's" not nebraska*) or (liverpool not (new south wales* or nsw)) or ("liverpool's" not (new south wales* or nsw)) or ((london not (ontario* or ont or toronto*)) or ("london's" not (ontario* or ont or toronto*)) or manchester or "manchester's" or (newcastle not (new south wales* or nsw)) or ("newcastle's" not (new south wales* or nsw)) or norwich or "norwich's" or nottingham or "nottingham's" or oxford or "oxford's" or peterborough or "peterborough's" or plymouth or "plymouth's" or portsmouth or "portsmouth's" or preston or "preston's" or ripon or "ripon's" or salford or "salford's" or salisbury or "salisbury's" or sheffield or "sheffield's" or southampton or "southampton's" or st albans or stoke or "stoke's" or sunderland or "sunderland's" or truro or "truro's" or wakefield or "wakefield's" or wells or westminster or "westminster's" or winchester or "winchester's" or wolverhampton or "wolverhampton's" or (worcester not (massachusetts* or boston* or harvard*)) or ("worcester's" not (massachusetts* or boston* or harvard*)) or (york not ("new york*" or ny or ontario* or ont or toronto*)) or ("york's" not ("new york*" or ny or ontario* or ont or toronto*))))).ti,ab,in,ad. (2957939)
**88**  (bangor or "bangor's" or cardiff or "cardiff's" or newport or "newport's" or st asaph or "st asaph's" or st davids or swansea or "swansea's").ti,ab,in,ad. (121751)
**89**  (aberdeen or "aberdeen's" or dundee or "dundee's" or edinburgh or "edinburgh's" or glasgow or "glasgow's" or inverness or (perth not australia*) or ("perth's" not australia*) or stirling or "stirling's").ti,ab,in,ad. (407021)
**90**  (armagh or "armagh's" or belfast or "belfast's" or lisburn or "lisburn's" or londonderry or "londonderry's" or derry or "derry's" or newry or "newry's").ti,ab,in,ad. (56781)
**91**  or/83-90 (4617446)
**92**  ((exp "arctic/ and antarctic"/) or exp oceanic regions/ or exp western hemisphere/ or exp africa/ or exp asia/) not (exp united kingdom/ or europe/) (3567731)
**93**  91 not 92 (4356800)
**94**  82 and 93 (10997)
**95**  limit 94 to dc=20230228-20240102 (2000)
**96**  (exp animal/ or animal experiment/ or nonhuman/) not (exp human/ or human experiment/) (7328293)
**97**  95 not 96 (1944)

**NIH Covid-19 Portfolio**: https://icite.od.nih.gov/covid19/search/

**Date of search**: 02/01/2024

All searches were date limited from 28/02/2023 to 02/01/2024

| **Search terms** | **Preprint server** | **No. results** |
| --- | --- | --- |
| “contact tracing” OR “contact investigation” OR “contact screening” OR “contact investigation” OR “contact testing” | ArXiv | 16 |
| (quarantin* or isolat*) OR (lockdown* or lock-down* OR shielding) | ArXiv | 16 |
| ("lateral flow" OR “COVID test” OR ”Coronavirus test” OR “home test”) | ArXiv | 3 |
| “physical distancing” OR “social distancing” OR “movement restriction” OR “mass gathering” OR “tiered restriction” | ArXiv | 18 |
| “environmental disinfection”~3 OR “surface disinfection”~3 OR “home disinfection”~3 OR “house disinfection”~3 OR “workplace disinfection”~3 OR deep clean* | ArXiv | 5 |
| “environmental cleaning”~3 OR “surface cleaning”~3 OR “home cleaning”~3 OR “house cleaning”~3 OR “workplace cleaning”~3 OR deep clean* | ArXiv | 5 |
| "hand santizer" OR "hand santiser" OR "hand washing" OR "hand hygiene" or handwashing | ArXiv | 0 |
| facemask or mask OR face covering | ArXiv | 0 |
| ("school closure" OR "workplace closure" OR "hospitality closure"~3 OR "home working") | ArXiv | 5 |
| “border health” OR “travel ban” OR “travel restriction” | ArXiv | 4 |
| (airflow* or “air flow” OR “air quality” or “indoor air” OR “air conditioning” OR “air filtration” OR HVAC) | ArXiv | 0 |
| “non pharmaceutical intervention” | ArXiv | 13 |
| **Search terms** | **Preprint server** | **No. results** |
| “contact tracing” OR “contact investigation” OR “contact screening” OR “contact investigation” OR “contact testing” | MedrXiv | 102 |
| (quarantin* or isolat*) OR (lockdown* or lock-down* OR shielding) | MedrXiv | 348 |
| ("lateral flow" OR “COVID test” OR ”Coronavirus test” OR “home test”) | MedrXiv | 82 |
| “physical distancing” OR “social distancing” OR “movement restriction” OR “mass gathering” OR “tiered restriction” | MedrXiv | 265 |
| “environmental disinfection”~3 OR “surface disinfection”~3 OR “home disinfection”~3 OR “house disinfection”~3 OR “workplace disinfection”~3 OR deep clean* | MedrXiv | 148 |
| “environmental cleaning”~3 OR “surface cleaning”~3 OR “home cleaning”~3 OR “house cleaning”~3 OR “workplace cleaning”~3 OR deep clean* | MedrXiv | 148 |
| "hand santizer" OR "hand santiser" OR "hand washing" OR "hand hygiene" or handwashing | MedrXiv | 29 |
| facemask or mask OR "face covering" | MedrXiv | 20 |
| ("school closure" OR "workplace closure" OR "hospitality closure"~3 OR "home working") | MedrXiv | 64 |
| “border health” OR “travel ban” OR “travel restriction” | MedrXiv | 73 |
| (airflow* or “air flow” OR “air quality” or “indoor air” OR “air condition*” OR “air filtration” OR HVAC) | MedrXiv | 12 |
| “non pharmaceutical intervention” | MedrXiv | 148 |
| **Search terms** | **Preprint server** | **No. results** |
| “contact tracing” OR “contact investigation” OR “contact screening” OR “contact investigation” OR “contact testing” | Research Square | 80 |
| (quarantin* or isolat*) OR (lockdown* or lock-down* OR shielding) | Research Square | 658 |
| ("lateral flow" OR “COVID test” OR ”Coronavirus test” OR “home test*”) | Research Square | 51 |
| “physical distancing” OR “social distancing” OR “movement restriction” OR “mass gathering” OR “tiered restriction” | Research Square | 434 |
| “environmental disinfection”~3 OR “surface disinfection”~3 OR “home disinfection”~3 OR “house disinfection”~3 OR “workplace disinfection”~3 OR deep clean* | Research Square | 273 |
| “environmental cleaning”~3 OR “surface cleaning”~3 OR “home cleaning”~3 OR “house cleaning”~3 OR “workplace cleaning”~3 OR deep clean* | Research Square | 273 |
| "hand santizer" OR "hand santiser" OR "hand washing" OR "hand hygiene" or handwashing | Research Square | 33 |
| facemask or mask OR "face covering" | Research Square | 27 |
| ("school closure" OR "workplace closure" OR "hospitality closure"~3 OR "home working") | Research Square | 107 |
| “border health” OR “travel ban” OR “travel restriction” | Research Square | 89 |
| (airflow* or “air flow” OR “air quality” or “indoor air” OR “air conditioning” OR “air filtration” OR HVAC) | Research Square | 17 |
| “non pharmaceutical intervention” | Research Square | 73 |

**CoronaCentral search**

https://coronacentral.ai/

Date of search: 02/01/2024

Searched for ‘NPI’ – filtered to virus type ‘Sars-Cov-2’ and Location: UK and Oxford (only UK locations available). Date limited from 24/02/23 to 02/01/24

4 results

**1B: Quality appraisal methodology for observational and interventional studies**

| **No.** | **Question** | **Status** |
| --- | --- | --- |
| 1 | Was the research question clearly stated? | General |
| 2 | Was the selection of the study subjects / patients free from bias? | Critical |
| 3 | Were the study groups comparable? | Critical |
| 4 | Was method of handling withdrawals described? | General |
| 5 | Was blinding used to prevent introduction of bias? | General |
| 6 | Were intervention / therapeutic regimens / exposure factor or procedure, and any comparison(s), described in detail? Were intervening factors described? | Critical |
| 7 | Were outcomes clearly defined and the measurements valid and reliable? | Critical |
| 8 | Was the statistical analysis appropriate for the study design and type of outcome indicators? | General |
| 9 | Are conclusions supported by results with biases and limitations taken into consideration? | General |
| 10 | Is bias due to study’s funding or sponsorship unlikely? | General |

This approach is based on the Quality Criteria Checklist. For each question, an answer of Yes, No or Unclear was given. Questions 2, 3, 6 and 7 are regarded as “critical” questions. Each paper was given a ranking of high, medium or low quality depending on the answers, as follows:

- **High quality:** All four critical questions (2, 3, 6 and 7) are answered yes; at least one general question is answered yes.
- **Medium quality:** At least two critical questions (2, 3, 6 or 7) are answered yes.
- **Low quality:** Any study not meeting the criteria for medium or high quality.

For the purpose of ranking the studies, an answer of “unclear” was treated as equivalent to “no”.

**1C: Quality appraisal methodology for modelling studies**

Adapted from quality appraisal tool developed by Burns et al, 2020†. For each of the questions (1-10) if this information was reported the reviewer would indicate whether there were no concerns or some concerns. If it was not reported, they would state “not reported”. They would use the applicability criteria and examples in the table to help them make an assessment.

| **Aspect** | **Questions** | **Application to the review** | **Example** |
| --- | --- | --- | --- |
| **Model structure** | 1. Are the structural assumptions transparent and justified? | 1. Assess whether all structural model assumptions are explicitly stated and whether the authors substantiate these assumptions either through theoretical reasoning or through prior knowledge from the literature. | • Description of model type and defining equations  • Comprehensible explanation of model variables and equations  • Description of features of the disease captured by the model, e.g. a randomly distributed incubation time  • Explanations of model structure implications by text or graphical representations visualising the simulation pathway, e.g. a scheme of the context being modelled  • Description of model limitations and simplifying assumptions. |
|  | 2. Are the structural assumptions reasonable given the overall objective, perspective and scope of the model? | 2. Consider whether the structural assumptions are consistent with what is known about the phenomenon of interest in the literature. In case of disagreement, assess to what extent these discrepancies undermine the overall validity of results and conclusions. **Mark as some concerns if the assumptions made by the model will affect the validity of model.** |  |
| **Input data** | 3. Are the input parameters transparent and justified? | 3. Assess whether the values of all input parameters are explicitly stated and whether the authors substantiate these values either through theoretical reasoning or through prior knowledge from the literature. | • Epidemiological characteristics known from other studies  • Inputs to data calibration algorithms  • Table with input parameters and probability distributions used for probabilistic modelling  • Explanation and discussion of choice of parameter values with appropriate citations |
|  | 4. Are the input parameters reasonable? | 4. Consider whether the input parameter values are consistent with what is known about the phenomenon of interest in the literature. In case of disagreement, assess to what extent these discrepancies undermine the overall validity of results and conclusions. **Mark as some concerns if study used aggregate data or used combination of individual and aggregate data.** |  |
| **Validation (external)** | 5. Has the external validation process been described? | 5. Assess whether there was a formal process of comparing the predictions of the model with 1) the data source that was used to build the model (dependent validation), 2) a data source that was not used to build the model, e.g. an independent country (independent validation) or 3) future values that did not intervene in model building (predictive validation). | • Calibration of SEIR model to case data (dependent validation)  • Prediction of a subset of observed data points based on training data set and comparison with validation data set (dependent validation)  • Prediction of data points of country/region that was not part of the model fitting and calibration process and comparison with observed data (independent validation)  • Prediction of future values that were not used in model building (predictive validation) |
|  | 6. Has the model been shown to be externally valid? | 6. Consider the extent to which model predictions agree with the data sources that were selected for the external validation process. |  |
| **Validation (internal)** | 7. Has the internal validation process been described? | 8. Consider the extent to which the results of the internal validation process indicate that the mathematical calculations are consistent with the model’s specifications. | • Application of the model on simulated data to establish that analyses work as intended  • Code review process conducted by authors or by an independent source to ensure correct implementation of mathematical structure  • Independent replication of model |
|  | 8. Has the model been shown to be internally valid? |  |  |
| **Uncertainty** | 9. Was there an adequate assessment of the effects of uncertainty? | 9. Consider whether the robustness of results to alternative input parameter values or model assumptions was assessed either by reporting the results of specific sensitivity analyses or through an app in which readers can themselves explore the effects of varying these model assumptions and input parameter values. **Mark as some concerns if no confidence intervals around estimates or prediction levels as this prevents evaluation on whether the effects of NPI were found by chance** | • Structural and parameter **sensitivity analyses** • Inherent stochasticity due to simulation nature of model  • Reporting of an app in which effects of input changes can be tracked  • Propagation of present uncertainties to outcomes  • Was the model **probabilistic**, i.e. were parameter values fixed or sampled from a distribution?  • Is **uncertainty** transparently reported, described and justified? |
| **Transparency** | 10. Was technical documentation, in sufficient detail to allow (potentially) for replication, made available openly or under agreements that protect intellectual property? | 10. Assess whether the description of the analyses (including model structure, input parameters, data sources and methods) is sufficiently detailed to allow for the replication of results. In particular, consider whether the code that was used to obtain the results is freely available and well documented. | • Description of model which is qualitatively extensive enough to allow for scrutiny of other researchers (e.g. supplementary material)  • Do authors encourage replication by clarifying a procedure to obtain code?  • Do the authors only refer to other, similar models for justification and detailed methodological description or do they provide their own documentation? |

†Burns J, Movsisyan A, Stratil JM, Coenen M, Emmert-Fees KM, Geffert K, et al. Travel‐related control measures to contain the COVID‐19 pandemic: a rapid review. Cochrane Database Syst Rev [Internet]. 2020 [cited 2024 Apr 13];(9). Available from: https://www.cochranelibrary.com/cdsr/doi/10.1002/14651858.CD013717/full

**1D: Decision algorithm for certainty of evidence**

**
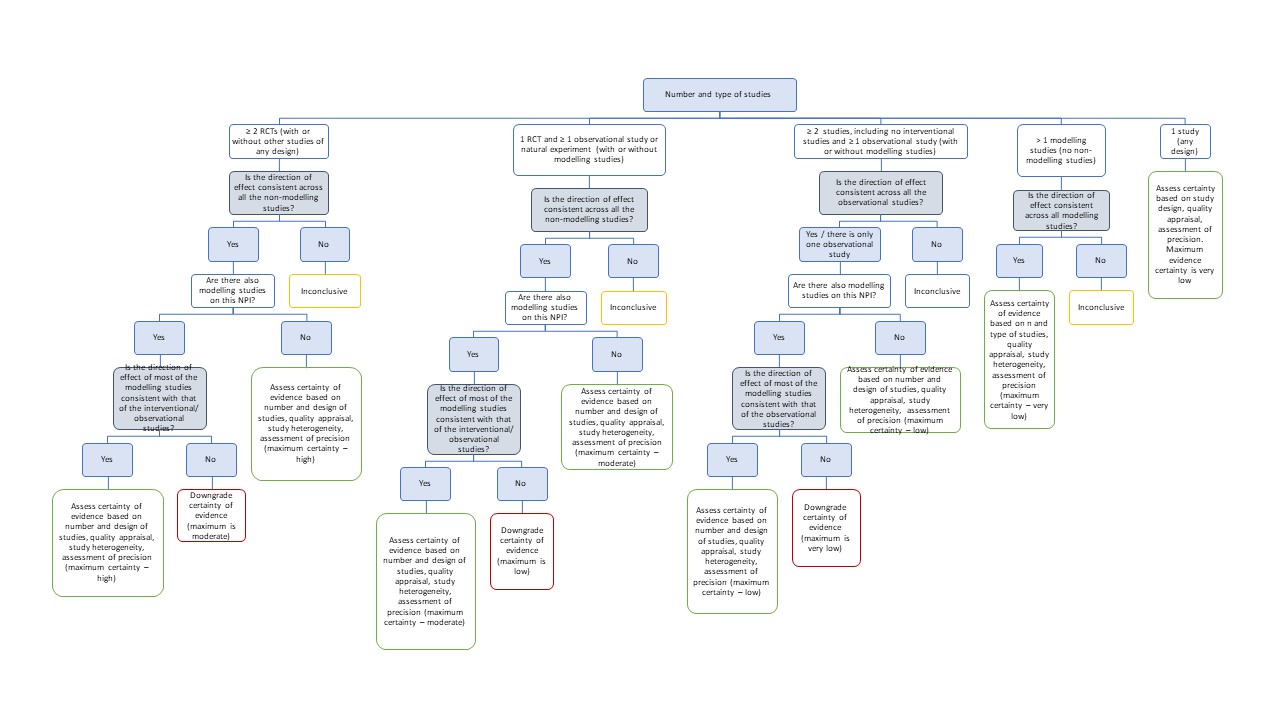
**

### Table 2A: Characteristics of included studies ordered alphabetically

| **Study ID** | **Study design and purpose** | **Scale/population and setting** | **Prediction/study period** | **NPI(s)** | **Comparator(s)** | **Outcome(s)** |
| --- | --- | --- | --- | --- | --- | --- |
| (Abernethy and Glass, 2022) (110) | Modelling (Age structured SEIR model)  Purpose: To explore hypothetical implementations of lockdowns including testing the influence of the duration and intensity of a lockdown | Scale: Northern Ireland whole population using ecological data  Setting: N/A | Not specified | Impact of intensity and duration of lockdown measures | No NPI(s) | COVID-19 transmission, hospital admissions, ICU admission and deaths |
| (Albi, Pareschi and Zanella, 2021) (94) | Modelling (SEIRD model)  Purpose: to describe the spread of the COVID-19 epidemic and assess the impact of selective relaxation of containment measures within several countries | Scale: UK whole population based on various sources of ecological data  Setting: N/A | 8th March to 1st June 2020 | Impact of relaxing lockdown measures (at different times; on school and work) | No NPI(s) | Infection rates |
| (Almagor and Picascia, 2020) (41) | Modelling (Agent-based SEIR model)  Purpose: to explore the effectiveness of the contact tracing app | Scale: Population-wide, based on 103,000 agents  Setting: Glasgow City | Not given | Contact tracing.  NHS COVID-19 app | Various scenarios, including a baseline of no testing and no app. | COVID-19 infections |
| (Alsing, Usher and Crowley, 2020) (54) | Modelling (Stochastic branching-process transmission model)  Purpose: To assess the efficacy of spatially targeted lockdown, or mass testing with isolation, as a complement to contact tracing and social distancing. | Scale: England, population wide, split by local authority areas based on collected ecological data  Setting: England and Wales | Not given | Asymptomatic testing (with isolation of cases)  Spatially targeted lockdowns  [N.B. in addition to existing policy of contact tracing and social distancing] | Contact tracing and social distancing only | COVID-19 infections  COVID-19 outbreaks |
| (Arnold et al., 2022) (103) | Modelling (Time varying counterfactual model)  Purpose: To estimate the reduction in confirmed COVID-19 cases and deaths that would have occurred in England by 1 June 2020 had public health interventions been introduced one or two weeks earlier. | Scale: England whole population using ecological data  Setting: N/A | 3rd March to1st June 2020 | Lockdown timing and duration | No NPI(s) | COVID-19 cases, deaths, case fatality ratios |
| (Aspinall et al., 2020) (85) | Modelling (Bayesian Belief network model)  Purpose: to develop a quick punctual model for enumerating infection hazard in primary schools | Scale: England  Setting: Primary schools using Department for Education data | Return to school June 2020 | Return to school in cohorts of children and teachers | Compared various partial or full return to school scenarios | Number of infected persons, number of schools with one or more infected person |
| (Banks et al., 2022) (112) | Modelling (Spatial ABM called Scotland Coronavirus transmission model)  Purpose: to estimate the impact of travel restrictions and transmission reduction on the spread of COVID-19, and to assess the impact of measures of deprivation to estimate its impact on COVID-19 related mortality. | Scale: Scotland whole population using ecological data  Setting: N/A | 9th March to 26th April 2020 | Impact of lockdown restrictions. Included area-based deprivation in model. | Baseline scenario no lockdown | COVID-19 transmission rate and mortality |
| (Bays et al., 2021) (122) | Modelling (Deterministic and stochastic with Monte Carlo simulation)    Purpose: To simulates the dynamics of a COVID-19 outbreak in a prison setting | Scale: Simulated model of  678 prisoners    Setting: Prison | Not specified | Shielding with cohorting and reverse cohorting | No shielding or cohorting | Infection rates, case attack rate, hospitalisations |
| (Bays et al., 2022) (70) | Modelling (Monte-Carlo time-varying model)  Purpose: To compare the benefits and risks of a single LFD negative result against multiple sequential LFD negative results to permit early release from isolation. | Scale: Population wide, based on simulated individual data  Setting: N/A | Not given | Test and Release strategy | Fixed period of isolation | Infectious cases released from isolation |
| (Biglarbeigi et al., 2021) (92) | Modelling (Estimate Time Varying Reproduction numbers)  Purpose: To study the effect of easing the restrictions by returning of different occupational groups to work | Scale: UK with breakdown by England, Scotland, Wales, and Northern Ireland  Setting: N/A | 10th February 2020 to 7th September 2020 | Return of different occupational groups to employment | Minimal contact in lockdown | R number |
| (Bittihn et al., 2021) (114) | Modelling (SIR)  Purpose: To determine the efficacy of regional containment strategies, where contact restrictions are triggered locally in individual regions upon crossing critical infection number thresholds | Scale: England whole population simulated model using ecological  Setting: N/A | Not specified | National and regional containment strategies | No lockdown | Days of effective national or regional lockdown and cross region leakiness |
| (Boldea, Cornea-Madeira and Madeira, 2023) (104) | Modelling (Dynamic intensity model)  Purpose: To assess the time varying effects of NPI(s) | Scale: England whole population using ecological data  Setting: N/A | 3rd May 2020 to 22nd January 2022 | National lockdown in comparison to regional lockdowns | No lockdown | Timing and steepness of lockdowns |
| (Brooks-Pollock et al., 2021) (42) | Modelling (Dynamic model)  Purpose: main purpose was to investigate re-opening of schools and also considered other NPIs related to limitations of contacts | Scale: UK population wide model with simulated data based on social contact survey in 2010 from 5,681 respondents  Setting: N/A | Not specified | Social distancing measures, school closures, face covering use, contact tracing, bubbles | Baseline scenario with only support bubbles allowed to mix. For contact tracing compared no contact tracing. | Secondary infections per case  Reproduction number, R |
| (Cheetham et al., 2021) (83) | Modelling (SEIR model)  Purpose: to determine level of social distancing compared and daily number of contacts to control COVID-19 spread | Scale: Northeast London simulated population based on ecological data at COVID-19 Dashboard  Setting: N/A | February - December 2020 | Limitations in social contacts | No limitations in social contacts | COVID-19 cases, deaths and hospitalisations related to number of contacts |
| (Chen and Qiu, 2021) (95) | Modelling (Dynamic panel SIR)  Purpose: To analyse the impact of different combinations of NPIs on the trajectory of the pandemic in 9 countries | Scale: UK whole population based on various sources of ecological data  Setting: N/A | 4th April to 9th August 2020 | Lockdown in combination with face covering, schools’ closure and quarantine | No NPI(s) | Cumulative number of cases |
| (Chin et al., 2021) (113) | Modelling (SIR)  Purpose: To compare effectiveness of the various NPIs for COVID-19 obtained from different SIR models in multiple countries | Scale: UK whole population based on various sources of ecological data  Setting: N/A | Two periods: (1) 4th Mar to 5th May 2020  (2) 4th Mar to 12th July 2020 | Lockdown and various NPIs that limit mobility | Other NPIs that limit mobility provided from 11 countries | Time varying R number |
| (Cuesta-Lazaro et al., 2021)  (30) | Modelling (Individual, ABM, called June)  Purpose: to explore the relative population-wide impacts of various NPIs and vaccination programmes implemented in secondary schools. | Scale: models potential COVID-19 outcomes across the whole UK population associated with introducing NPIs in secondary schools.  Setting: secondary schools | 10 July 2021 to 1 February 2022 | Use of face coverings  Ventilation  Isolation of contacts (class quarantine)  Social distancing  (NPIs are modelled as reducing the intensity of contacts between agents). | Pre-pandemic levels of contact intensity | Cumulative COVID-19 deaths |
| (Davies et al., 2020)  (39) | Modelling (SEIR)  Purpose: To assess the potential impact of NPIs for mitigating COVID-19 burden before the introduction of vaccines. | Scale: UK population-wide model with simulated data on the 66.4 million UK population.  Setting: N/A | 29 January 2020 – 31 December 2021 | Physical distancing  Isolation of cases  School closures alone and in combination with other NPIs  Shielding for people 70 years and older, compared with no interventions as well as multiple NPIs | No NPIs | COVID-19 cases, deaths, peak hospital beds required, time to peak cases |
| (Davies et al., 2021) (84) | Modelling (Dynamic compartmental)  Purpose: to examine tiered restriction and alternative lockdown strategies including school closures | Scale: England, Northern Ireland and Wales whole population using collected individual and aggregate level data  Setting: N/A | October 2020 to March 2021 | School closures with circuit breaker/fire breaker lockdowns  Tiered restrictions and circuit/fire breaker strategies in Wales and Northern Ireland | Baseline counterfactual scenario | Transmission, hospital admissions, deaths |
| (Davis et al., 2021) (43) | Modelling (Branching process model)  Purpose: To investigate the feasibility of contact tracing and the conditions under which it is effective | Scale: Based on simulated individual data, applied to the general population in UK  Setting: N/A | Not given | Contact tracing | Higher adherence to, and coverage of, contact tracing compared to lower adherence and/or coverage | Reproduction number, R  Adherence |
| (Didelot et al., 2023) (105) | Modelling (Bayesian latent process model)  Purpose: To estimate the impact of NPIs for COVID-19 in England | Scale: England whole population, simulated model based on ecological data  Setting: N/A | Not specified | Lockdown (November 2020) | No NPI(s) | R number over time |
| (Dong et al., 2022) (106) | Modelling (Recurrent Neural network)  Purpose: To understand the efficacy of control measures on the national and local development of the pandemic | Scale: England whole population by local authority areas  Setting: N/A | Week 46 to 51 2020 | Universal lockdown compared to various restrictions | Various combinations of lockdown and restrictions | Effect on predicted cases for LA with highest predicted cases |
| (Donnat et al., 2021) (31) | Modelling (nonparametric k-nearest neighbour)  Purpose: To provide risk metrics for live public events. | Scale: models’ potential transmission at an indoor public event and the impact on community transmission rates.  Setting: public building - Royal Albert Hall (5,000 simulated concert attendees) | 20 August 2020, 21 January 2021, 20 March 2021 | Use of face coverings | Varying levels of compliance | COVID-19 infection rate |
| (Drakesmith et al., 2022) (55) | Modelling (Bayesian time series model)  Purpose: To assess the impact of the whole-area testing pilot in Merthyr Tydfil by modelling the number of onward infections prevented. | Scale: Population-wide, based on individual collected data  Setting: Merthyr Tydfil and Lower Cynon Valley, Wales (people over 11 only) | 21 Nov 2020 to 3 Jan 2021 (infections) or 10 Feb 2021 (healthcare burden) | Asymptomatic testing | No testing | COVID-19 infections  Hospital admissions  ICU admissions  COVID-19 deaths |
| (Endo et al., 2021) (44) | Modelling (Branching process model)  Purpose: To explore the effectiveness of combining ‘backward’ tracing with conventional ‘forward’ tracing on SARS-CoV-2 transmission | Scale: Based on simulation of two index cases from a transmission tree  Setting: N/A | Not given | “Backward” contact tracing, in addition to forward contact tracing | Forward contact tracing only | Secondary infections per case |
| (Fairbanks et al., 2023) (26) | Cross-sectional  Purpose: To explore the relationship between social activities / contact behaviours and infection risk. | Population: 62 adult staff and students  Setting: Nottingham university | October 2020 – March 2021 | Use of face coverings  Physical distancing  Hand hygiene | No NPI | Positive COVID-19 test result |
| (Farkas and Chatzopoulos, 2021) (65) | Modelling (SEAIR model)  Purpose: To assess the impact of (self-)quarantine of symptomatic infectious individuals on disease dynamics. | Scale: UK population-wide, based on simulated ecological data  Setting: N/A | Mar to Jul 2020 | Isolation of cases | Weaker adherence to isolation of cases | COVID-19 infections |
| (Ferretti et al., 2021) (75) | Modelling (Integrated model)  Purpose: To evaluate the effectiveness of daily LFD antigen testing for traced contacts of COVID-19 cases, compared to quarantine. | Scale: Population wide, based on collected ecological data  Setting: N/A | Not given | Test and release strategies | Isolation of contacts | COVID-19 infections |
| (Filipe et al., 2023) (117) | Case control  Purpose: To evaluate the effect of the shielding programme on mortality by linkage  of health care records | Population: those over the age of 70 years registered at a General practice in April 2020 those shielding and not shielding identified through health records (n=57,713)  Setting: City of Liverpool | April 2020 to June 2021 | Shielding for those over 70 all the time and during high-risk periods | Non shielding | All-cause mortality (hazards ratio), HR for effect of shielding |
| (Findlater et al., 2022) (40) | Natural experiment  Purpose: To investigate the impact of delays in contact tracing (because of a system error) on COVID-19 transmission, hospitalisations, and mortality. | Population: 15,285 participants who submitted details to contact tracing system.  Setting: England (nation-wide contract tracing system error) | 30 September to 5 October 2020 | Contact tracing | Delayed contact tracing | Risk of infection of contacts, Hospital admissions, COVID-19 deaths at 28 and 60 days |
| (Fitz-Simon et al., 2023) (32) | Modelling (linear regression)  Purpose: to investigate the potential impact of counterfactual scenario in which face coverings were used by 90 % of the population from the start of the pandemic. | Scale: Northern Ireland population-wide model  Setting: N/A | 12 March to 10 August 2020 | Use of face coverings | Observed population face covering compliance | COVID-19 hospitalisation |
| (Francis et al., 2023) (27) | Cross-sectional  Purpose: Explore use of self reported NPIs and COVID-19 infection | Population: UK-wide  General population  27,758 respondents aged 16 years plus.  Setting: online survey recruited via social media, community organisations, academic institutions, and General Practitioners | November 2020 to May 2021 | Use of face coverings  Physical distancing  Hand hygiene  Respiratory hygiene  Cleaning surfaces | No NPI | COVID-19 infection |
| (Fyles et al., 2021) (45) | Modelling (Household structured branching process model)  Purpose: To investigate how contact tracing and isolation might affect the growth rate and probability of epidemic extinction | Scale: Based on simulated and collected ecological data  Setting: UK households | May to June 2020 | Contact tracing | Different levels of physical distancing | COVID-19 infections |
| (Galanis et al., 2021) (96) | Modelling (SEIR)  Purpose: To assess the influence of NPIs on physical distancing | Scale: UK whole population based on various sources of ecological data  Setting: N/A | Not specified | Lockdown | Baseline no lockdown | R number |
| (Ghoroghi, Rezgui and Wallace, 2022) (33) | Modelling (non-SIR ABM; computational fluid dynamics model)  Purpose: To model transmission in a public building and to identify protective factors (individual and environmental) | Scale: models’ potential transmission in a public building.  Setting: public building – foyer of The Forum, Cardiff University (62 simulated individuals) | No information | Use of face coverings  Hand hygiene  Ventilation | No NPI | Mean probability of secondary cases |
| (Goscé et al., 2020) (56) | Modelling (modified SEIR)  Purpose: To test the impact of borough specific restrictions of movement on transmission within borough and city of London | Scale: London, whole population within London and its boroughs  Setting: N/A | 547 days starting from 9 Mar 2020 | Citywide lockdown with and without use of testing, face covering and contact tracing, asymptomatic testing, isolation of cases, shielding of people older than 60 years | Various scenarios in combination with other NPIs | Ratio of cumulative deaths, R number |
| (Grassly et al., 2020) (46) | Modelling (Mathematical model)  Purpose: To investigate the potential impact of different testing and isolation strategies on transmission of SARS-CoV-2. | Scale: Population wide; based on simulated ecological data  Setting: UK | (not given) | Contact tracing.  Asymptomatic testing (of health workers)  Isolation of cases | No NPIs | Reproduction number, R |
| (He et al., 2021) (47) | Model type: Three stage agent-based simulation.  Purpose: To quantify how the effectiveness and resource requirements of Test-Trace-Isolate [TTI] systems vary | Scale: UK population wide, based on 40,162 agents  Setting: N/A | June to August 2020 | Contact tracing | Various scenarios including more or less strict packages of NPIs | Reproduction number, R |
| (Heald et al., 2021) (34) | Model type: non-SIR-based.  Purpose: To model the potential impact of face covering use in public transport and retail settings. | Scale: models potential COVID-19 outcomes across the whole UK population associated with face covering use in retail outlets and on public transport.  Setting: retail outlets and public transport. | 24 July – 22 October 2020 | Use of face coverings | No face covering (pre-mandated face covering) | COVID-19 infections, hospitalisations, and deaths |
| (Hill et al., 2021a) (52) | Model type: IBN.  Purpose: to assess the impact of workplace-targeted NPIs against spread of SARS-CoV-2 among a population of workers | Scale: UK simulated model based on ONS working sector data and census data  Setting: N/A | Not specified | Contact tracing.  Working from home, partial return to work, COVID-Secure workplace | Various scenarios compared against each other | Peak number of cases, size and duration of outbreak, days spent in isolation |
| (Hill et al., 2021b) (48) | Model type: Individual SEIR based network model.  Purpose: To assess the impact of adherence to isolation requirements and engagement with test and trace; adopting a policy of strict room isolation for on-campus residents displaying symptom; and mass testing among students at UK universities. | Scale: Simulated population of 25,000 students  Setting: a UK university | Autumn term 2020 | Contact tracing.  Asymptomatic testing  Isolation of cases | Lower adherence to the same NPIs | COVID-19 infections  Adherence |
| (Hill, 2023) (76) | Model type: SEIR model.  Purpose: To model impact of 5 Christmas bubble scenarios in England December 2020 | Scale: UK population wide model using ecological ONS data to create simulated population of 100,000 households.  Setting: N/A | December 2020 to January 2021 | Christmas bubble scenarios with household mixing | Five alternative scenarios with scenario A as no change and scenario C as original plan for Christmas 2020 in England | Number and percentage increase cumulative in infections |
| (Hilton et al., 2022) (78) | Model type: SEIR model.  Purpose: to produce an age and household structured model | Scale: England whole population simulated population based on 2011 England and Wales census  Setting: N/A | Not specified | Impact of household mixing, temporary relaxation of NPI and out of household isolation | No NPI(s) | Transmission rate |
| (Hinch et al., 2022) (107) | Model type: ABM.  Purpose: to understand spatial-temporal characteristics of the COVID-19 epidemic | Scale: England upper-tier local authorities. The model used data to simulate a population of 1 million people.  Setting: N/A | Not specified | Various lockdown scenarios related to duration and timing of lockdowns | Actual lockdown in December 2020 and January 2021 | Cases and deaths avoided |
| (Jani et al., 2021) (118) | Cohort  Purpose: To compare those classified as high risk and advised to shield and those moderate and low risk in terms of COVID-19 outcomes | Population: West of Scotland, using linkage of health records with shielding classified by NHS Greater Glasgow and Clyde criteria (n=1,315,071)  Setting: N/A | March to May 2020 | Shielding for those at highest risk and advice for those at moderate risk | General restrictions for people at low risk | Relative risk of COVID-19 infection, hospital/ICU admission, mortality |
| (Jarvis et al., 2020) (93) | Cross sectional  Purpose: To survey contact patterns and compliance with physical distancing measures | Population: Adults recruited through market research company by email  UK (n=1356)  Setting: online survey in UK | 24 to 27 March 2020 | Lockdown | Prior to lockdown | Pre and post lockdown intervention ratio |
| (Julliard, Shi and Yuan, 2023) (115) | Model type: SIR.  Purpose: To explore whether geographically targeted lockdown (i.e., working from home restrictions as implemented in UK during the first lockdown) might have been as effective at containing the epidemic as full-scale lockdown. | Scale: 32 London boroughs, modelled using ecological and mobility data  Setting: N/A | Not specified | Timing and targeting approach to lockdowns | First lockdown implemented in London | Number of cases averted and reduction in total cases |
| (Kaiser, Kretschmer and Leszczensky, 2021) (91) | Model type: ABM.  Purpose: To examine the effectiveness of different cohorting strategies to curb the spread of SARS-CoV-2 in schools | Scale: England, simulated population created using ecological data and survey data from school children 2010 to 2011 from 4 countries.  Setting: School aged children aged 14-15 years | Not specified | School intervention Effects of cohorting compared to no cohorting within school environment | No cohorting | Proportion of outbreaks, proportion of infected students, proportion quarantined |
| (Kamiya et al., 2023) (111) | Model type: SIR.  Purpose: To see if earlier lockdowns would have resulted in fewer COVID-19 hospitalisations. | Scale: Northern Ireland whole population using ecological data  Setting: N/A | 5th March 2020 to end of February 2021 | Lockdowns that were 7 or 14 days earlier than those implemented | Three lockdowns implemented | Cumulative number of hospitalisation |
| (Keeling et al., 2021a) (97) | Model type: SEIR.  Purpose: to model the 2-week precautionary break on trajectory of COVID-19 infections | Scale: UK whole population based on UK individual and aggregate level data  Setting: N/A | 1st Oct 2020 to 1st Jan 2021 | 2-week lockdowns (e.g., circuit breakers) | Without 2-week lockdown | COVID-19 infections, hospitalisations, and deaths |
| (Keeling et al., 2021b) (86) | Model type: SEIR.  Purpose: to explore different approaches to opening schools | Scale: UK whole population using collected aggregate and individual level data  Setting: N/A | 1st June to 22nd July 2020 | Return to school re-opening with variation in class size and year returning to school | Continuation of school closures | Secondary infections, clinical cases, R number |
| (Kucharski et al., 2020) (49) | Model type: Individual level mathematical model.  Purpose: To understand what combination(s) of control measures (including contact tracing, self-isolation and contact restrictions) are effective in reducing COVID-19 transmission. | Scale: Uses collected ecological data from the BBC Pandemic dataset and published studies of COVID-19 contact tracing  Setting: UK | Not given | Contact tracing.  Asymptomatic testing  Isolation of cases | No NPIs | COVID-19 infections |
| (Kumari et al., 2021) (119) | Cross sectional  Purpose: To investigate the impact of receiving a shielding letter on symptom reporting | Population: participants of Understanding Society longitudinal UK study asked to complete a monthly web-survey. Only those who completed every survey included (n=13,754)  Setting: N/A | April to July 2020 | Household where someone received a letter advising to shield for 12 weeks and those shielding | No shielding | Probability of reporting COVID-19 symptoms or odds ratio for positive test (in supplementary materials) |
| (Kunzmann et al., 2021) (57) | Modelling (Agent-based non-SIR model)  Purpose: To assess the impact of different test and isolation schemes on containment of outbreaks and school days missed in a primary school setting. | Scale: Primary school children; based on simulated individual data  Setting: Primary schools in England | A six-week period in 2020 or 2021 | Asymptomatic testing  Test and Release strategies | Isolation of symptomatic cases only | COVID-19 infections  Time lost to education |
| (Laydon et al., 2021) (116) | Modelling (Bayesian hierarchical model)  Purpose: To measure the effects of the tier system on the COVID-19 pandemic in the UK between the first and second national lockdowns | Scale: UK whole population, at lower tier local authorities (310)  Setting: N/A | 1st July 2020 to 5th November 2020 | Tiered restrictions, 2 and 3 compared to 1 or no restrictions | Before tier system was introduced | Real time R number, percentage reduction in transmission |
| (Leng et al., 2021) (79) | Modelling (Stochastic simulation model)  Purpose: to estimate the impact of different social bubbles | Scale: England whole population simulated population using England and Wales 2011 census data.  Setting: N/A | Not specified | Contact clustering and social bubbles | Counterfactual scenarios not including bubbles | Transmission rate and mortality |
| (Leng et al., 2022a) (58) | Modelling (Approximate Bayesian computation fitted to stochastic IBN model)  Purpose: To assess the potential impact of a range of isolation and testing strategies on transmission and absences of secondary school students in England over half-term and a 7-week term. | Scale: England, department of educational data  Setting: Secondary schools aged 11-16 years | 31 Aug 2020 to 23 May 2021 | School intervention School bubbles with various testing strategies | Each scenarios compared against each other | Transmission, infection rate, school absence |
| (Leng et al., 2022b) (53) | Model type: Epidemiological model.  Purpose: To explore the general impacts of different notification windows on SARS-CoV-2 transmission. | Scale: Based on collected ecological data of NHS app use  Setting: England and Wales | Before and after 2 August 2021 | NHS COVID-19 app (5-day notification window) | NHS COVID-19 app (2-day notification window) | Reproduction number, R  Adherence |
| (Leng et al., 2022c) (59) | Model type: Approximate Bayesian computation fitted to stochastic IBM.  Purpose: to estimate the proportion of infections in secondary school pupils due to pupil-to-pupil transmission | Scale: England, department of educational data  Setting: Secondary schools aged 11-16 years | 31st August to 23rd May 2021 | School intervention School bubbles, mass testing, serial contact testing | Various different strategies compared against each other | Testing rate and number of absences from school |
| (Love et al., 2022a) (72) | Randomised controlled trial  Purpose: To investigate whether a test-and-release strategy (daily testing with LFDs + no isolation if negative) is as effective as 10-day self-isolation of contacts for minimising transmission of COVID-19. | Population:  26,123 participants in test-to-release group; 23,500 participants in self-isolation group  Setting: England (nation-wide, recruited via the contact tracing system) | 29 Apr to 28 Jul 2021 | Test and release strategies | Isolation of contacts | COVID-19 infections  Adherence  Acceptability |
| (Love et al., 2022b) (74) | Case-control study  Purpose: To evaluate the acceptability and feasibility of a test-to-release strategy for contacts of COVID-19 cases | Population:  882 participants in daily testing group, 878 participants in declined daily testing group  Setting: England (nation-wide, recruited via the contact tracing system) | 11-23 Dec 2020 and 4-12 Jan 2021 | Test and release strategies | Isolation of contacts | COVID-19 infections  Adherence |
| (Lovell-Read, Shen and Thompson, 2022) (77) | Model type: SEIR model  Purpose: to model impacts of school closures, workplace closures and broader social distancing policies | Scale: UK population wide model simulated population  Setting: N/A | Not specified | Combination of lockdown, school closures, social distancing, and surveillance | Compared with each other and combinations of NPIs | Probability of local outbreaks |
| (Lucas et al., 2021) (50) | Model type: Branching process model  Purpose: To examine which aspects of contact tracing adherence should be prioritised, exploring the trade-offs between engagement with self-reporting and adherence to self-isolation. | Scale: Based on simulated ecological data  Setting: UK | Not given | Contact tracing | Different degrees of adherence to contact tracing | COVID-19 infections  Adherence |
| (Marchant et al., 2022) (29) | Cross-sectional  Purpose: To examine association between COVID-19 positive cases within primary school setting with school-based mitigation measures | Population: 353 teaching staff at 59 primary schools in Wales completing questionnaires (within 15 out of 22 local authorities)  Setting: primary schools | 9th October to 16th December 2020 | Face covering use, physical distancing,  teaching indoors and outdoors, availability of clubs, outdoor teaching | No NPI | COVID-19 cases |
| (Makris, 2021) (98) | Model type: SIR  Purpose: to contributes to the understanding of the impact of government intervention on social distancing and the epidemic by studying various lockdown episodes | Scale: UK whole population simulated population  Setting: N/A | 24th March to 30th May | Lockdown | Various social distancing measures | Infection induced fatality rates |
| (Mégarbane, Bourasset and Scherrmann, 2021) (99) | Model type: SIR  Purpose: study aimed to compare lockdown- attributed effects on SARS-CoV-2 epidemic progression in nine countries with various lockdown scenarios. | Scale: UK whole population simulated model based on WHO data  Setting: N/A | Not specified | Lockdown compared to other strategies | Comparison of lockdowns between countries | Maximum rate of new cases, rate of regression |
| (Miller et al., 2022) (29) | Model type: Quantitative microbial risk assessment  Purpose: To model the impact of environmental factors and NPIs on the infectious dose received by passengers. | Scale: models the infectious dose received by passengers travelling on public transport with an infected person.  Setting: public transport -London underground train carriage (28 – 176 simulated passengers, representing varying levels of carriage occupancy). | Not specified | Use of face coverings    Ventilation | Range of environmental and behavioural scenarios (mask compliance, number of passengers, ventilation rate) | COVID-19 infectious dose |
| (Mintram et al., 2022) (108) | Model type: ABM  Purpose: To compare baseline scenarios and periodic lockdowns with and without vaccination | Scale: England whole population, simulated population based on 2012 Health survey for England  Setting: N/A | Not specified | Periodic lockdowns with and without vaccination | Baseline scenario no lockdown | Hospital admissions |
| (Moore et al., 2021) (35) | Model type: Individual-based stochastic model  Purpose: To assess the efficacy of lateral flow devices (LFDs) to screen for asymptomatic infection or "test to release" scenarios. | Scale: School and university populations; based on collected ecological data  Setting: UK schools and universities | Not given | Use of face coverings  Ventilation  Asymptomatic testing | Either: face mask use and/or ventilation (in schools)  Or: lower rates of testing (in higher education) | COVID-19 infections |
| (Muegge et al., 2023) (109) | Model type: Bayesian spatial-temporal random effects model  Purpose: To investigate spatio-temporal trends in COVID-19 mortality risks following the implementation of 3 national lockdowns in England | Scale: England whole population using ecological data  Setting: N/A | Not specified | Three lockdowns March to May 2020, November 2020 and Jan to Mar 2021 | No NPI(s) | COVID-19 mortality |
| (Munday et al., 2021a) (88) | Model type: contact matrices  Purpose: estimate the effect on R number of schools reopening | Scale: UK whole population collected individual data  Setting: N/A | Between January to March 2021 (prior to opening on 8th March 2021) | Return to school with partial or full reopening | Baseline R value when schools ere closed | R number |
| (Munday et al., 2021b) (87) | Model type: Transmission probability network  Purpose: To evaluate the risk of transmission between schools and pupil households under various school re-opening scenarios. | Scale: Schools in England (21,583 schools)  Setting: state funded primary and secondary schools | Not specified | Return to school with reopening allowing different year groups in primary and secondary schools | No NPI(s) | Transmission between schools and pupil households |
| (Nadim, Ghosh and Chattopadhyay, 2021) (66) | Model type: SEAIR model with additional compartments  Purpose: To explore control studies that can significantly reduce the COVID-19 outbreak in the UK. | Scale: UK population-wide, based on collected ecological data  Setting: N/A | 21 Nov to 4 Dec 2020 | Isolation of cases  Isolation of contacts | Same NPIs with differing levels of infectiousness | COVID-19 infections |
| (Novakovic and Marshall, 2022) (36) | Model type: Change Point Detection into an ABM  Purpose: To estimate the effectiveness of face coverings, compared to a counterfactual scenario of 0% population face covering use from 10 August to 1 October 2020 | Scale: Northern Ireland population-wide model  Setting: N/A | 10 August – 1 October 2020 | Use of face coverings | Observed population face covering compliance | COVID-19 cases |
| (Panovska-Griffiths et al., 2020) (90) | Model type: Stochastic ABM (Covasim)  Purpose: Predict the impact of possible strategies for reopening schools to all students in the UK from September 2020 | Scale: UK whole population using ecological data for UK  Setting: N/A | 21st January 2020 to 31st December 2020 | Return to school Partial and full reopening of school with testing and contact tracing | No NPI(s) | Daily cumulative number of infection and deaths, R number |
| (Panovska-Griffiths et al., 2021)(38) | Model type: Stochastic ABM (Covasim)  Purpose: To explore whether extending mandatory face covering use to secondary school students, alongside public transport and retail, would have reduced/ prevented the resurgence of COVID-19 from September 2020 | Scale: UK whole population using ecological data for UK  Setting: secondary schools | 21 January to 28 August 2020 | Use of face coverings | Face coverings in community only | Daily cumulative number of infection and deaths, R number, proportion of symptomatic people who would have to have sought testing to avoid second wave in autumn 2020. |
| (Panovska-Griffiths et al., 2022) (89) | Model type: Stochastic ABM (Covasim)    Purpose: Understand the effect of various scenarios of partial or full reopening of schools with ongoing broader social lockdown measures | Scale: UK whole population using ecological data for UK  Setting: N/A | 22 February 2021 to 20 April 2021 | Partial and full reopening of schools with full and partial lockdown | No NPI(s) | Daily cumulative number of infection and deaths, R number |
| (Post et al., 2021) (100) | Model type: SEIR  Purpose: To examine how effective contact rate (mean number of daily contacts for an infectious individual) in seven European countries evolved over a period of various government restrictions. | Scale: UK whole population using ecological data from John Hopkins University dashboard  Setting: N/A | Not specified | Lockdown March 2020 | No NPI(s) | Effective contact rate |
| (Quilty et al., 2021) (68) | Model type: Stochastic individual-based model Based on  Purpose: To evaluate the effect of different quarantine measured and testing strategies on reducing onward transmission from secondary infections. | Scale: UK population wide, based on simulated individual data  Setting: N/A | Not given | Isolation of contacts  Test and release strategies | Isolation of symptomatic cases only | COVID-19 infections |
| (Quilty, Pulliam and Pearson, 2022) (71) | Model type: Simulated logistic regression model  Purpose: To evaluate test to release policies against conventional fixed duration isolation policies in terms of excess days of infectiousness, days saved, and tests used. | Scale: Sample populations of 10,000 people in UK  Setting: N/A | Not given | Test and release strategies | 10-day isolation of contacts | Infectious cases released from isolation |
| (Ruget et al., 2021) (82) | Model type: stochastic network meta-population model  Purpose: to estimate the impact of different social bubbles | Scale: Hebridean islands in Scotland within islands and travel to and from mainland Scotland  Setting: N/A | Not specified | Limitation in social contacts, reduction in movement from mainland, lockdown scenarios | Baseline scenarios no restrictions and 6 daily contacts | Number of infections |
| (Sandmann et al., 2020) (60) | Model type: Static decision-analytic model  Purpose: To explore the impact of different testing strategies for key workers, on duration of absence from work and risk of transmission to others | Scale: 1,000 key workers, based on simulated data  Setting: N/A | Not given | Asymptomatic testing | Different testing scenarios | COVID-19 infections  Time lost to employment |
| (Silva et al., 2023) (61) | Model type: Individual-based SEIR model with contact layers  Purpose: To explore the impact of regular asymptomatic testing on the peak and total number of infections in an emerging COVID-19 wave. | Scale: 100,000 agents, based on simulated individual data  Setting: UK town | August to September 2020 | Asymptomatic testing | No asymptomatic testing | COVID-1 infections |
| (Smith, Yates and Ashby, 2022) (121) | Model type: SEIR    Purpose: To evaluate the effect of shielding the most vulnerable and allowing infection to spread among lower risk individuals | Scale: England, whole population, simulated population  Setting: N/A | Not specified | Shielding of vulnerable cases in two scenarios (perfect and imperfect) | No shielding | Mortality rate |
| (Snooks et al., 2023) (120) | Cohort  Purpose: To describe the demographics of the shielding population and compare COVID-19 outcomes between shielded and non shielded populations | Population: C20 cohort which include general population living in Wales on 23^rd^ March 2020. Study used health records with those shielding identified using clinical categories  (n= 3,203,800)  Setting: N/A | March 2020 to March 2021 | Shielding for those at highest risk | Non shielding | COVID-19 testing rate, proportion of positive tests and proportion of known infection, COVID-19 hospital admissions and deaths |
| (Sonabend et al., 2021) (80) | Model type: SEIR  Purpose: to quantify the impact of each of four steps of the roadmap to lifting restrictions | Scale: England whole population using ecological data  Setting: N/A | 21st June 2021 to 1st January 2022 | Examined the impact of four steps of the roadmap out of lockdown | Various counterfactual scenarios | COVID-19 cases, hospital admissions and deaths |
| (Stocks et al., 2023) (51) | Model type: Data-driven egocentric network model  Purpose: To evaluate how social behaviours change in a university setting under COVID-19 restrictions, and to analyse the effectiveness of contact tracing in this setting. | Scale: Based on collected individual data  Setting: a UK university | June 2020 to February 2021 | Contact tracing (with or without social distancing) | No contact tracing | COVID-19 infections |
| (van Bunnik et al., 2021) (101) | Model type: SIR  Purpose: To investigate the exit strategy for the first lockdown | Scale: UK whole population simulated using ecological data  Setting: N/A | 1 year after lockdown (dates not specified) | Lockdown then relaxation of restrictions | Pre-lockdown | Transmission rates |
| (Violato, Violato and Violato, 2021) (102) | Model type: SEM  Purpose: To assess the impact of lockdown measures on the spread of COVID-19 | Scale: UK whole population using ecological  Setting: N/A | Not specified | Lockdown | No lockdown | Mortality and infection rates |
| (Warne et al., 2021) (62) | Model type: Simple compart-mental SEAIR model  Purpose: To evaluate the impact of different testing strategies on SARS-CoV-2 cases and numbers of students in isolation over the term. | Scale: University population; based on collected ecological data  Setting: UK university | Not specified | Asymptomatic testing | No asymptomatic testing | Reproduction number, R |
| (Wells et al., 2020) (67) | Model type: Individual-based stochastic difference equation SEAIR model  Purpose: To explore the outcomes of different control strategies to contain the size of the COVID-19 epidemic, in the context of urban-rural gradients in prevalence and susceptibility. | Scale: County-wide, based on individual simulated data  Setting: Four counties in South-West Wales | Not specified | Isolation of contacts  Isolation of cases | Different combinations of NPIs | COVID-19 infections |
| (Whitfield et al., 2023) (63) | Model type: Stochastic agent-based network model  Purpose: To estimate the efficacy of different workplace interventions with a model particularly tailored to the home-delivery sector. | Scale: A company’s workforce; based on collected individual data  Setting: Workplace interactions in a home delivery company | July to August 2020 and May to June 2021 | Asymptomatic testing, physical distancing, office staff working from home, driver pairings | No NPIs | COVID-19 infections |
| (Woodhouse et al., 2022) (64) | Model type: Time-varying SEUQR model  Purpose: To quantify projected infection patterns within primary school classrooms, and related uncertainties. | Scale: Model based on 10 primary schools, each consisting of 4 classrooms with 30 pupils and one teacher  Setting: Primary schools | First term of the 2021 school year | Asymptomatic testing  School intervention Bubble quarantine with and without testing | Bubble quarantine and no mitigation measures | COVID-19 infections in pupils  Time lost to education |
| (Ying and O’Clery, 2021) (37) | Model type: Dynamic ABM  Purpose: To study of the respiratory droplet transmission due to customers coming into close contact with one another. | Scale: Supermarkets and retail stores in UK  Setting: Retail store | Not specified | Restriction in customer number, arrival rate, one-way system, face coverings and combinations of NPIs | No NPI(s) | Number of infectious plateaus, number of infections, chance of infection |
| (Young et al., 2021) (73) | Randomised controlled trial  To investigate whether a test-and-release strategy for school-based contacts of COVID-19 cases would be as effective as 10-day self-isolation in controlling COVID-19 transmission, while resulting in greater school attendance. | Population: 201 schools (randomisation took place at school level)  Setting: Secondary schools and further education colleges in England | 19 April to 27 June 2021 | Test and release strategies | Isolation of contacts | COVID-19 infections |
| (Ziauddeen, Subramaniam and Gurdasani, 2021) (81) | Model type: Bayesian  Purpose: To model infections and deaths under various scenarios | Scale: England whole population using ONS death data  Setting: N/A | Not specified | Easing of lockdown measures from May to July 2020 | Lockdown when R was estimated to be 0.75 | Number of excess cases and deaths |
| (Zhang et al., 2022) (69) | Model type: Modified SEIR model  Purpose: To evaluate the impact of stay-at-home and quarantine measures on COVID-19 spread in four cities that experienced large-scale outbreaks in the spring of 2020: Wuhan, New York, Milan, and London. | Scale: City wide, using collected ecological data  Setting: London | 24 March to 19 June 2020 | Isolation of contacts | No isolation of contacts | COVID-19 infections |

ABM = Agent Based Model, IBN = Individual based network, ONS= Office of National Statistics, SIR = Susceptible, Infected and Recovered, SEIR = Susceptible, Exposed, Infected and Recovered, SEUQR = Susceptible, Exposed, Unwell, Quarantined, Recovered N/A = not applicable

**2B: Table of quality appraisal of observational and interventional studies ordered alphabetically**

*Questions 2, 3, 6 and 7, coloured blue, are regarded as “critical” questions, 4 of which had to be answered “Yes” for a study to be deemed of high quality and at least 2 answered “Yes” for moderate quality*1

| **Study ID** | **Design** | **1** | **2** | **3** | **4** | **5** | **6** | **7** | **8** | **9** | **10** | **Ranking** |
| --- | --- | --- | --- | --- | --- | --- | --- | --- | --- | --- | --- | --- |
| (Fairbanks et al., 2023) | Cross-sectional | Y | N | Y | N | N | Y | Y | Y | Y | Y | Moderate |
| (Filipe et al., 2023) | Case-control | Y | Y | Y | Y | N | Y | Y | Y | Y | Y | High |
| (Findlater et al., 2022) | Natural experiment | Y | Y | Y | Y | N | Y | Y | Y | Y | Y | High |
| (Francis et al., 2023) | Cross-sectional | Y | N | Y | N | U | Y | Y | Y | Y | Y | Moderate |
| (Jani et al., 2021) | Cohort | Y | Y | Y | N | N | Y | Y | Y | Y | Y | High |
| (Jarvis et al., 2020) | Cross-sectional | Y | N | U | N | N | Y | Y | Y | Y | Y | Moderate |
| (Kumari et al., 2021) | Cross-sectional | Y | N | Y | U | N | N | Y | Y | Y | Y | Moderate |
| (Love et al., 2022a) | RCT | Y | Y | Y | Y | N | Y | Y | Y | Y | Y | High |
| (Love et al., 2022b) | Case-control | Y | Y | N | N | U | Y | U | N | Y | Y | Moderate |
| (Marchant et al., 2022) | Cross-sectional | Y | N | Y | U | N | Y | N | Y | Y | Y | Moderate |
| (Snooks et al., 2023) | Cohort | Y | Y | Y | Y | Y | Y | Y | Y | N | Y | High |
| (Young et al., 2021) | RCT | Y | Y | Y | Y | N | Y | Y | Y | Y | N | High |

*Legend: 1= research question, 2= subject selection, 3= comparable groups, 4 = withdrawals, 5= blinding, 6= provisional of detail, 7= outcomes, 8 = statistical analysis, 9 = conclusions, 10= bias, , Y=yes, N= No, U= unclear*

**2C: Table of quality appraisal of modelling studies listed alphabetically**

*Labels indicate whether there were some or no quality concerns or whether key elements were reported*

| **Study ID** | **1** | **2** | **3** | **4** | **5** | **6** | | **7** | **8** | **9** | **10** |
| --- | --- | --- | --- | --- | --- | --- | --- | --- | --- | --- | --- |
| (Abernethy and Glass, 2022) | No | Some | No | Some | Not reported | | | Reported | Some | No | No |
| (Albi, Pareschi and Zanella, 2021) | No | No | No | Some | Not reported | | | Reported | Some | Some | Some |
| (Almagor and Picascia, 2020) | No | Some | No | Some | Reported | | Some | Reported | Some | Some | No |
| (Alsing, Usher and Crowley, 2020) | No | Some | No | Some | Not reported | | | Not reported | | Some | No |
| (Arnold et al., 2022) | No | Some | No | Some | Not reported | | | Not reported | | No | No |
| (Aspinall et al., 2020) | No | No | No | Some | Not reported | | | Reported | No | No | No |
| (Banks et al., 2022) | No | Some | No | Some | Reported | Some | | Reported | Some | Some | No |
| (Bays et al., 2021) | No | Some | No | Some | Not reported | | | Not reported | | Some | Some |
| (Bays et al., 2022) | No | No | No | No | Not reported | | | Not reported | | No | Some |
| (Biglarbeigi et al., 2021) | No | No | No | Some | Not reported | | | Reported | No | No | No |
| (Bittihn et al., 2021) | No | No | No | Some | Not reported | | | Reported | Some | Some | No |
| (Boldea, Cornea-Madeira and Madeira, 2023) | No | Some | No | Some | Not reported | | | Not reported | | Some | Some |
| (Brooks-Pollock et al., 2021) | No | Some | No | Some | Not reported | | | Not reported | | No | No |
| (Cheetham et al., 2021) | No | Some | No | Some | Not reported | | | Not reported | | Some | No |
| (Chen and Qiu, 2021) | Some | Some | No | Some | Not reported | | | Not reported | | Some | Some |
| (Chin et al., 2021) | No | Some | No | Some | Not reported | | | Reported | Some | No | No |
| (Cuesta-Lazaro et al., 2021) | No | No | No | Some | Not reported | | | Not reported | | No | No |
| (Davies et al., 2020) | No | No | No | No | Not reported | | | Not reported | | No | No |
| (Davies et al., 2021) | No | Some | No | No | Reported | Some | | Reported | No | No | No |
| (Davis et al., 2021) | No | No | No | No | Not reported | | | Not reported | | Some | No |
| (Didelot et al., 2023) | No | No | No | No | Not reported | | | Not reported | | Some | No |
| (Dong et al., 2022) | No | Some | No | Some | Reported | Some | | Reported | No | Some | No |
| (Donnat et al., 2021) | No | No | No | No | Reported | No | | Reported | Some | Some | No |
| (Drakesmith et al., 2022) | No | No | No | No | Not reported | | | Not reported | | No | No |
| (Endo et al., 2021) | No | No | No | No | Not reported | | | Not reported | | Some | No |
| (Farkas and Chatzopoulos, 2021) | No | Some | No | No | Not reported | | | Not reported | | Some | Some |
| (Ferretti et al., 2021) | No | No | No | No | Not reported | | | Not reported | | Some | No |
| (Fitz-Simon *et al.*, 2023) | No | No | No | No | Not reported | | | Not reported | | Some | No |
| (Fyles et al., 2021) | No | No | No | No | Not reported | | | Reported | No | No | No |
| (Galanis et al., 2021) | No | Some | No | Some | Not reported | | | Not reported | | Some | Some |
| (Ghoroghi, Rezgui and Wallace, 2022) | No | No | No | Some | Not reported | | | Not reported | | Some | Some |
| (Goscé et al., 2020) | No | No | No | No | Not reported | | | Not reported | | Some | No |
| (Grassly et al., 2020) | No | No | No | Some | Not reported | | | Not reported | | No | No |
| (He et al., 2021) | No | No | No | Some | Not reported | | | Reported | Some | No | No |
| (Heald *et al.*, 2021) | No | No | No | Some | Not reported | | | Not reported | | Some | No |
| (Hill et al., 2021a) | No | No | No | Some | Not reported | | | Not reported | | No | No |
| (Hill et al., 2021b) | No | No | No | No | Not reported | | | Not reported | | No | No |
| (Hill, 2023) | No | No | No | Some | Not reported | | | Not reported | | No | No |
| (Hilton et al., 2022) | No | No | No | Some | Not reported | | | Not reported | | Some | No |
| (Hinch et al., 2022) | No | Some | Some | Some | Reported | Some | | Reported | No | No | No |
| (Julliard, Shi and Yuan, 2023) | No | No | No | No | Reported | Some | | Reported | No | Some | No |
| (Kaiser, Kretschmer and LeszczenskY, 2021) | Some | No | Some | Some | Not reported | | | Not reported | | No | No |
| (Kamiya et al., 2023) | No | No | No | No | Not reported | | | Reported | No | Some | Some |
| (Keeling et al., 2021a) | No | No | No | Some | Not reported | | | Reported | No | Some | No |
| (Keeling et al., 2021b) | No | No | No | Some | Not reported | | | Reported | No | No | Some |
| (Kucharski et al., 2020) | No | No | No | Some | Not reported | | | Not reported | | Some | No |
| (Kunzmann et al., 2021) | No | No | No | No | Not reported | | | Reported | No | No | No |
| (Laydon et al., 2021) | No | Some | No | Some | Not reported | | | Reported | No | No | No |
| (Leng et al., 2021) | No | Some | No | Some | Not reported | | | Not reported | | Some | No |
| (Leng et al., 2022a) | No | No | No | No | Not reported | | | Not reported | | No | No |
| (Leng et al., 2022b) | No | Some | No | Some | Not reported | | | Reported | Some | Some | Some |
| (Leng et al., 2022c) | No | Some | No | Some | Not reported | | | Not reported | | No | No |
| (Lovell-Read, Shen and Thompson, 2022) | No | Some | Some | Some | Not reported | | | Not reported | | Some | Some |
| (Lucas et al., 2021) | No | Some | No | No | Not reported | | | Not reported | | No | No |
| (Makris, 2021) | No | Some | No | Some | Not reported | | | Reported | Some | Some | No |
| (Mégarbane, Bourasset and Scherrmann, 2021) | No | Some | No | Some | Not reported | | | Reported | No | No | Some |
| (Miller *et al.*, 2022) | No | Some | No | Some | Not reported | | | Not reported | | Some | Some |
| (Mintram et al., 2022) | No | No | No | No | Not reported | | | Reported | No | No | No |
| (Moore et al., 2021) | No | No | No | Some | Not reported | | | Not reported | | No | No |
| (Muegge et al., 2023) | No | No | No | Some | Not reported | | | Reported | No | No | No |
| (Munday et al., 2021a) | No | No | No | No | Not reported | | | Not reported | | No | No |
| (Munday et al., 2021b) | No | No | No | Some | Not reported | | | Not reported | | No | No |
| (Nadim, Ghosh and ChattopadhYaY, 2021) | No | No | Some | No | Not reported | | | Reported | No | Some | No |
| (Novakovic and Marshall, 2022) | No | No | No | Some | Reported | | Some | Reported | No | No | No |
| (Panovska-Griffiths et al., 2020) | No | No | No | Some | Not reported | | | Not reported | | Some | No |
| (Panovska-Griffiths et al., 2021) | No | No | No | Some | Not reported | | | Not reported | | No | No |
| (Panovska-Griffiths et al., 2022) | No | No | No | Some | Reported | Some | | Reported | No | No | No |
| (Post et al., 2021) | No | Some | No | Some | Not reported | | | Not reported | | No | No |
| (Quilty et al., 2021) | Some | Some | No | Some | Not reported | | | Not reported | | Some | No |
| (Quilty, Pulliam and Pearson, 2022) | Some | Some | Some | Some | Not reported | | | Not reported | | Some | Some |
| (Ruget et al., 2021) | No | No | No | Some | Not reported | | | Reported | Some | Some | No |
| (Sandmann et al., 2020) | Some | Some | Some | Some | Not reported | | | Not reported | | No | No |
| (Silva et al., 2023) | No | Some | No | No | Reported | | No | Not reported | | No | No |
| (Smith, Yates and Ashby, 2022) | No | No | No | No | Not reported | | | Not reported | | No | No |
| (Sonabend et al., 2021) | No | No | No | Some | Reported | Some | | Reported | No | No | No |
| (Stocks et al., 2023) | None | None | None | Some | Not reported | | | Not reported | | Some | None |
| (van Bunnik et al., 2021) | No | No | No | Some | Not reported | | | Not reported | | No | No |
| (Violato, Violato and Violato, 2021) | No | Some | No | Some | Not reported | | | Not reported | | Some | No |
| (Warne et al., 2021) | No | Some | No | Some | Not reported | | | Not reported | | No | No |
| (Wells et al., 2020) | No | Some | No | No | Not reported | | | Reported | No | Some | No |
| (Whitfield et al., 2023) | No | Some | No | No | Not reported | | | Reported | No | Some | No |
| (Woodhouse et al., 2022) | No | No | No | No | Not reported | | | Reported | No | Some | No |
| (Ying and O’Clery, 2021) | No | No | No | Some | Not reported | | | Reported | Some | No | No |
| (Ziauddeen, Subramaniam and Gurdasani, 2021) | No | Some | No | Some | Not reported | | | Reported | Some | Some | Some |
| (Zhang et al., 2022a) | None | Some | None | Some | Not reported | | | Reported | Some | Some | None |

*Legend: 1 = transparent structural assumptions, 2= reasonable structural assumptions, 3= transparent input parameters, 4 = reasonable input parameters, 5= external validation described 6= model shown to be externally valid, 7= internal validation described, 8 = model shown to be internally valid, 9 = assessment of certainty, 10 = transparency.*
